# Intratumoral oncolytic herpes virus G47∆ for residual or recurrent glioblastoma: a phase 2 trial

**DOI:** 10.1038/s41591-022-01897-x

**Published:** 2022-07-21

**Authors:** Tomoki Todo, Hirotaka Ito, Yasushi Ino, Hiroshi Ohtsu, Yasunori Ota, Junji Shibahara, Minoru Tanaka

**Affiliations:** 1 Division of Innovative Cancer Therapy, Advanced Clinical Research Center, and Department of Surgical Neuro-Oncology, The Institute of Medical Science, The University of Tokyo, Tokyo, Japan; 2 Department of Data Science, National Center for Global Health and Medicine in Japan, Tokyo, Japan; 3 Leading Center for the Development and Research of Cancer Medicine, Juntendo University, Tokyo, Japan; 4 Department of Pathology, The Institute of Medical Science, The University of Tokyo, Tokyo, Japan; 5 Department of Pathology, Kyorin University School of Medicine, Tokyo, Japan

**Keywords:** CNS cancer, Translational research

## Abstract

This investigator-initiated, phase 2, single-arm trial primarily assessed the efficacy of G47∆, a triple-mutated, third-generation oncolytic herpes simplex virus type 1, in 19 adult patients with residual or recurrent, supratentorial glioblastoma after radiation therapy and temozolomide (UMIN-CTR Clinical Trial Registry UMIN000015995). G47Δ was administered intratumorally and repeatedly for up to six doses. The primary endpoint of 1-yr survival rate after G47∆ initiation was 84.2% (95% confidence interval, 60.4–96.6; 16 of 19). The prespecified endpoint was met and the trial was terminated early. Regarding secondary endpoints, the median overall survival was 20.2 (16.8–23.6) months after G47∆ initiation and 28.8 (20.1–37.5) months from the initial surgery. The most common G47∆-related adverse event was fever (17 of 19) followed by vomiting, nausea, lymphocytopenia and leukopenia. On magnetic resonance imaging, enlargement of and contrast-enhancement clearing within the target lesion repeatedly occurred after each G47∆ administration, which was characteristic to this therapy. Thus, the best overall response in 2 yr was partial response in one patient and stable disease in 18 patients. Biopsies revealed increasing numbers of tumor-infiltrating CD4^+^/CD8^+^ lymphocytes and persistent low numbers of Foxp3^+^ cells. This study showed a survival benefit and good safety profile, which led to the approval of G47∆ as the first oncolytic virus product in Japan.

## Abstract (web summary)

Results from a pivotal single-arm phase 2 trial show that the repeated intratumoral administration of the oncolytic herpes virus G47∆ in residual or recurrent glioblastoma exhibits survival benefit and a safe profile.

## Main

Glioblastoma has a poor prognosis with a median overall survival (OS) of 20.9 months despite the current Stupp regimen of radiotherapy plus temozolomide together with tumor-treating fields (TTF)^1^. This regimen with TTF is the current standard-of-care for newly diagnosed glioblastoma in the United States, but its recommendation level as a standard-of-care varies among countries outside the United States. None of the current therapies can yet prevent recurrence of glioblastoma, not to mention cure it, so nonconventional therapeutic approaches are needed especially for recurrent cases.

G47Δ is a triple-mutated, third-generation oncolytic herpes simplex virus type 1 (HSV-1) constructed by deleting the *α47* gene and overlapping *US11* promoter from parental G207, a second-generation oncolytic HSV-1 with deletions in both copies of the *γ34.5* gene and an inactivation of the *ICP6* gene^2^. G47Δ shows greater tumor-specific replication capability and cytopathic effects than G207 but retains a high safety profile^3,4^. G47Δ was confirmed safe in the first-in-human (FIH) trial when administered intratumorally, two doses within 2 weeks, to patients with recurrent glioblastoma^5^. Preclinical studies show that G47∆ exhibits efficacy via two mechanisms: (1) an immediate effect via virus replication and direct oncolytic activity; and (2) a delayed effect via induction of specific antitumor immunity^2^.

In Japan, national health insurance allows patients to choose any government-approved treatment at any institution and at relatively low cost^6^. In clinical studies for lethal diseases in Japan, setting a noncurable standard-care control arm would not be accepted and also would be unethical, especially if the study was an academia-initiated, research grant-supported drug development such as this. Further, because G47∆ needs to be administered by surgery, blinding treatment arms and allowing sham surgery on randomly selected patients would not be considered acceptable in Japan.

This phase 2 trial is, therefore, designed as a single-arm trial to evaluate the efficacy of G47Δ in patients with glioblastoma that was residual or recurred after initial therapy of surgery, radiation and temozolomide.

## Results

**Extended Data Fig. 1:**
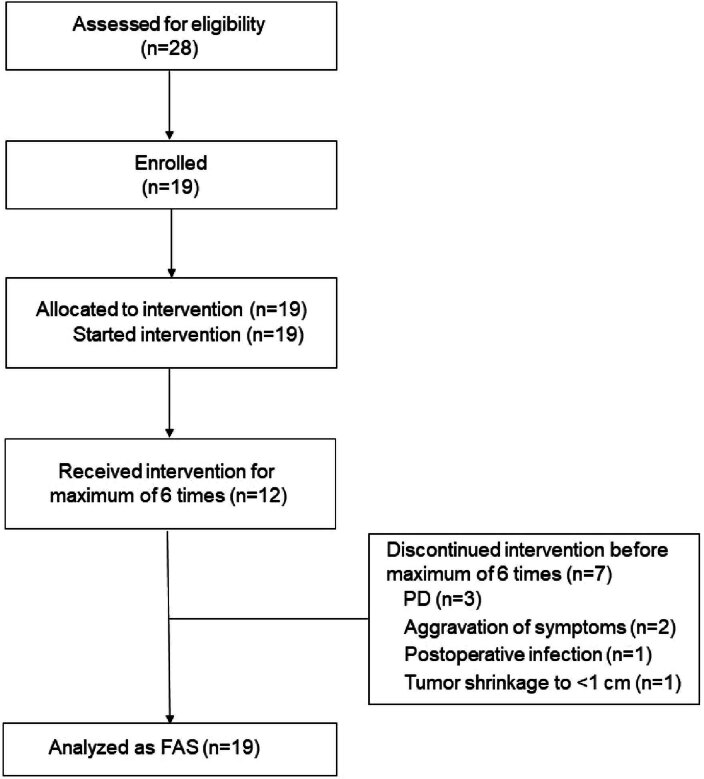
Patient disposition. Of 28 patients who gave informed consent, 19 patients who matched the eligibility criteria were enrolled and composed the full analysis set (FAS).

The interim analysis in 13 patients showed a 1-yr survival rate after G47∆ initiation of 92.3% (95% confidence interval (95% CI), 64.0–99.8). Compared with the preset control value of 15%, the O’Brien–Fleming boundary was crossed for the planned interim report (*Z*_0_ = 7.806). This confirmed that the prespecified primary endpoint of this study was met and, therefore, the trial was terminated early by the recommendation of the Independent Data Monitoring Committee and as predetermined by the protocol. Of 28 patients who gave informed consent, 19 patients who matched the eligibility criteria had been enrolled and composed the full analysis set (FAS) (Extended Data Fig. 1). Demographics and baseline clinical characteristics of these 19 enrolled patients are shown in Table 1.

**Table 1:** Baseline demographic and clinical characteristics

Pt no.	Sex/ age	KPS	Tumor location	Surgery	Radiation (Gy/fr)	Chemotherapy other than TMZ	HSV-1 IgM/IgG^a^	Ki-67 (%)	IDH1	MGMT promoter MSP	MGMT expression	Rec/ Res	Time from initial surgery to G47Δ (months)	Tumor area Baseline (mm^2^)	MET-PET L/N	Steroids	
																Before G47Δ	New or increased dose during G47Δ treatment
1	M/54	90	R/Fr	GTR	50.4/28	−	0.23/<2.0	4	wt	NA	+	1st	12.4	375.2	1.71	−	−
2	M/49	90	R/Fr	PR	60/30	−	0.09/<2.0	5	wt	NA	−	Res	3.0	220.0	1.50	−	−
3	F/63	80	L/Fr	PR	60/30	−	0.10/15.1	5	wt	NA	+	Res	3.4	661.4	3.62	−	−
4	M/69	90	L/T	GTR	60/30	−	0.19/2.7	15	wt	unmet	+	1st	8.7	176.9	1.78	−	−
5	M/46	100	R/Fr	GTR	50.4/28	BEV	0.46/51.4	6	wt	unmet	−	1st	14.1	554.2	1.95	−	Hydrocortisone (500 mg ×1 dose, 5 times)
6	F/25	70	L/P	PR	60/30	BEV, BCNU wafer	0.13/<2.0	7	mt	unmet	+	2nd	23.5	696.6	2.15	−	−
7	M/51	90	R/T	PR	60/30	−	0.44/<2.0	40	wt	NA	−	Res	3.0	440.7	2.50	−	−
8	M/73	70	R/Fr	PR	60/30	−	0.18/123.0	30	wt	NA	+	1st	5.3	1,386.5	1.02	Prednisolone 10 mg d^−1^	−
9	M/46	100	R/Fr	GTR	60/30	BCNU wafer, IFNβ	0.18/<2.0	8	wt	NA	−	1st	24.9	122.4	1^b^	−	−
10	M/28	100	L/Fr	GTR	60/30	BEV, BCNU wafer	0.24/<2.0	30–40	mt	NA	−	1st	9.4	141.4	1.60	−	−
11	M/53	80	R/T	GTR	60/30	BCNU wafer	0.20/<2.0	2	mt	NA	−	1st	7.4	416.2	1.35	−	−
12	M/59	70	L/Fr	Biopsy	60/30	BEV	0.31/2.3	40	wt	met	−	1st	4.2	519.9	1.38	−	Dexamethasone (19.8 mg d^−1^ to 6.6 mg d^−1^ ×6 d, 1 time)
13	F/53	70	R/Fr	PR	60/30	BEV, BCNU wafer	0.45/105.0	30	mt	met	−	Res	4.1	873.1	1.86	−	−
14	M/42	80	Splenium	PR	60/30	−	0.19/<2.0	50	wt	NA	+	1st	10.1	1,832.2	2.27	−	Dexamethasone (19.8 mg d^−1^ to 13.2 mg d^−1^ ×4 d, 1 time); prednisolone (30 mg d^−1^ to 15 mg d^−1^ ×6 d, 1 time)
15	M/65	80	L/P	Biopsy	60/30	BEV	0.23/164.0	15	wt	NA	+	Res	4.4	652.8	1.54	−	Betamethasone (24 mg d^−1^ to 6 mg d^−1^ ×6 d, 1 time)
16	M/45	100	R/Fr	PR	60/30	BCNU wafer	0.10/<2.0	50	mt	NA	−	Res	3.1	2,306.7	2.64	−	−
17	M/68	90	R/T	PR	60/30	−	0.18/220.0	20	wt	NA	+	Res	3.9	291.5	2.39	−	−
18	F/43	80	L/P	Biopsy	60/30	BEV	0.30/216.0	10	wt	NA	−	Res	3.8	513.5	2.01	−	Prednisolone (30 mg d^−1^ ×7 d, 1 time)
19	M/35	80	R/Fr	PR	59.4/33	−	0.14/<2.0	90	mt	NA	−	Res	3.6	778.5	1.27	−	−

**Abbreviations:** F, female; M, male; KPS, Karnofsky Performance Scale; R, right; L, left; Fr, frontal lobe; T, temporal lobe; P, parietal lobe; GTR, gross total resection; PR, partial resection; TMZ, temozolomide; BEV, bevacizumab; BCNU wafer, carmustine wafer; IFNβ, interferon-β; HSV-1, herpes simplex virus type 1; IDH1, isocitrate dehydrogenase 1; mt, mutant type; wt, wild type; MGMT, methylguanine methyltransferase; MSP, methylation-specific PCR; met, methylated; unmet, unmethylated; NA, not available; Rec, recurrent; Res, residual; 1st, first recurrence; 2nd, second recurrence; MET, methionine; PET, positron emission tomography; L/N, lesion-to-normal ratio.

^a^1 d before first therapy.

^b^L/N could not be measured, because there was no increased uptake corresponding to the target lesion.

GTR is defined as >95% of the tumor removed assessed by the surgeon. Resection other than GTR is either PR or biopsy as judged by the surgeon.

Immunohistochemistry for MGMT expression: –, <10%; +, ≥10% and <50%; ++, ≥50%.

### Magnetic resonance imaging (MRI)-guided stereotactic injection

A total of 97 stereotactic surgeries were performed in 19 enrolled patients. Twelve patients (63.2%) received G47Δ administration the maximum of six times (Table 2). For the others, G47∆ treatment was discontinued for meeting one of the treatment discontinuation criteria, as follows: progressive disease judgment by increase in target lesion size (*n* = 3), aggravation of symptoms (*n* = 2), clinical judgment by investigator (postoperative infection) (*n* = 1) and shrinkage of the target lesion to <1 cm in diameter (*n* = 1).

**Table 2:** Summary of patient outcomes

Patient no.	Sex/ age	Number of G47∆ doses	Time to progression (d)^a^	Best response	OS (d)		Treatment after progression	Outcome	Autopsy	Cause of death
					After initial surgery	After 1st G47Δ therapy				
1	M/54	6	258	SD	863	485	Bevacizumab after reoperation (GTR)	Dead	+	Tumor progression
2	M/49	6	588	SD	1,748	1,657	Extended field SRS	Dead	+	Tumor progression
3	F/63	6	762^b^	SD	1,044	941	−	Dead	−	Tumor progression
4	M/69	5	115	SD	891	626	Bevacizumab	Dead	−	Tumor progression
5	M/46	6	133	SD	1,310	882	Bevacizumab after reoperation (GTR) Nimustine	Dead	+	Tumor progression
6	F/25	3	43	SD	897	181	Bevacizumab	Dead	−	Tumor progression
7	M/51	2	64	SD	489	398	Bevacizumab	Dead	−	Tumor progression
8	M/73	6	294	SD	817	655	Extended field SRS; bevacizumab	Dead	−	Tumor progression
9	M/46	6	1,696^b^	SD	2,716^c^	1,960^c^	−	Stable	NA	NA
10	M/28	4	79	SD	1,452	1,166	Extended field SRS	Dead	−	Tumor progression
11	M/53	6	1,584^b^	SD	2,073^c^	1,848^c^	−	Stable	NA	NA
12	M/59	6	139	SD	596	469	Bevacizumab	Dead	−	Tumor progression
13	F/53	6	573^b^	SD	709	583	−	Dead	+	Not related to the disease
14	M/42	4	78	SD	543	236	Bevacizumab	Dead	−	Tumor progression
15	M/65	6	142	SD	569	434	Bevacizumab	Dead	−	Tumor progression
16	M/45	6	163	SD	699	606	Extended field SRS; bevacizumab	Dead	+	Tumor progression
17	M/68	2	127	PR	1,554^c^	1,435^c^	Bevacizumab after reoperation (partial resection)	Stable	NA	NA
18	F/43	5	108	SD	244	127	−	Dead	−	Tumor progression
19	M/35	6	331	SD	667	556	Bevacizumab; spinal irradiation	Dead	−	Tumor progression

**Abbreviations:** F, female; M, male; PR, partial response; SD, stable disease; GTR, gross total resection; SRS, stereotactic radiosurgery (radiotherapy).

^a^Including the date of first administration.

^b^Tumor progression occurred after initial cut-off date for judgment of outcome.

^c^Patient remained alive at most recent observation date (1 March 2022) used to analyze survival.

Extent of tumor resection at reoperation: GTR is defined as >95% of the tumor removed assessed by the surgeon.

### Efficacy

**Fig. 1:**
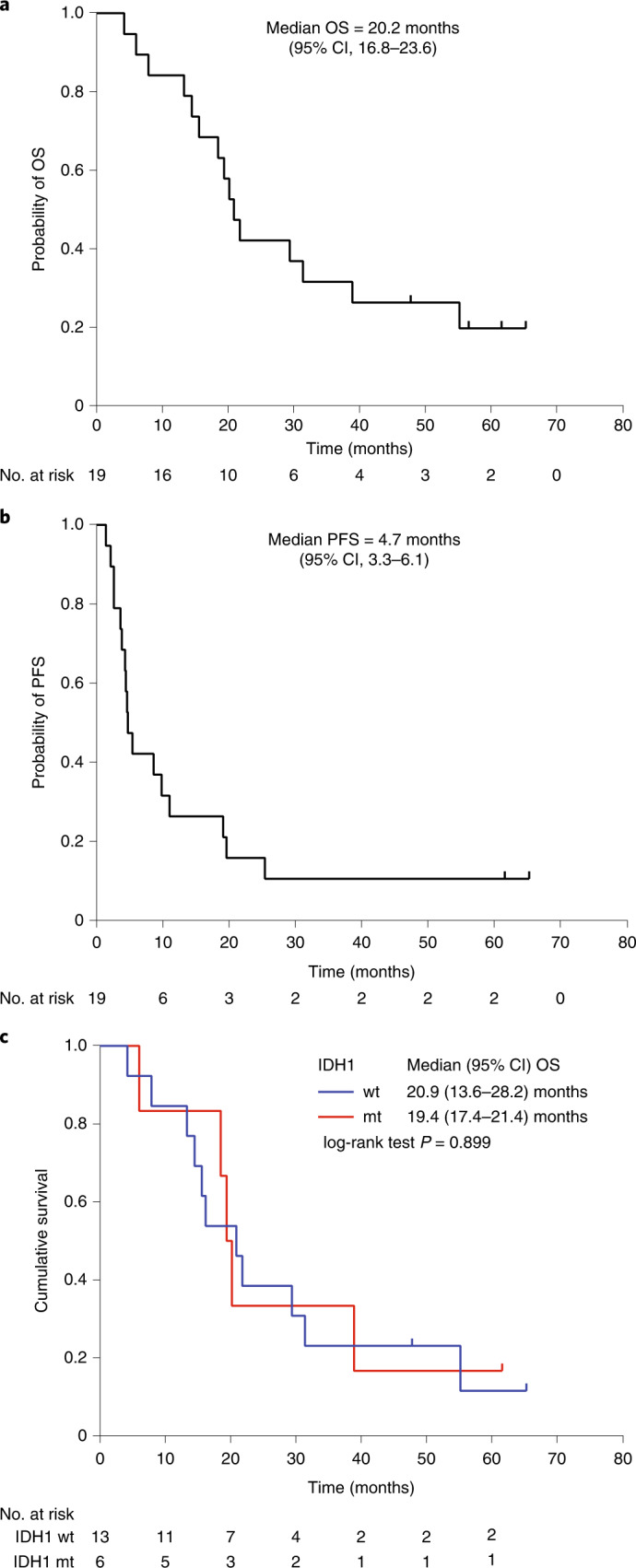
Kaplan-Meier curves after G47Δ initiation. **a**–**c**, Kaplan–Meier curves for OS after G47∆ initiation (**a**), PFS after G47∆ initiation (**b**) and OS based on IDH1 status after G47Δ initiation (**c**). The data were analyzed and Kaplan–Meier curves created on 1 March 2022. Source data

**Extended Data Fig. 2:**
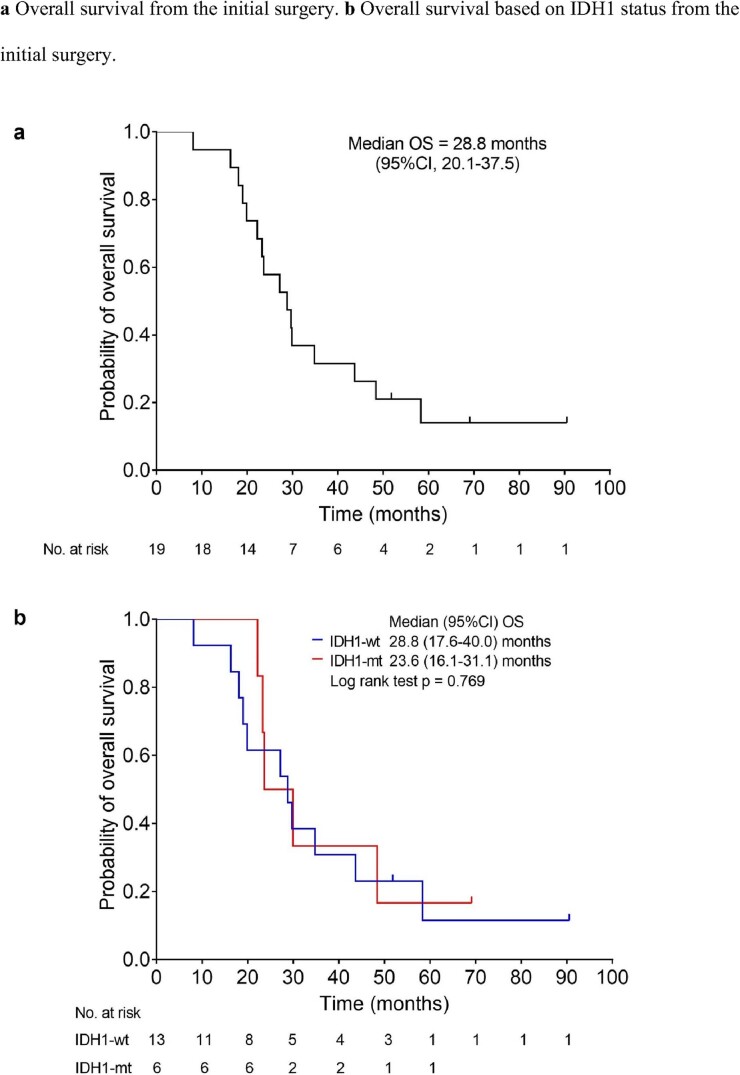
Kaplan-Meier curves from the initial surgery. **a** Overall survival from the initial surgery. **b** Overall survival based on IDH1 status from the initial surgery. Source data

The primary endpoint was the 1-yr survival rate and the secondary endpoints included OS and progression-free survival (PFS), all after G47∆ initiation. For the FAS population, the 1-yr survival rate after G47∆ initiation was 84.2% (95% CI, 60.4–96.6). The median OS was 20.2 (16.8–23.6) months after G47∆ initiation (Fig. 1a and Table 2), and the median PFS was 4.7 (3.3–6.1) months after G47Δ initiation (Fig. 1b). As an exploratory endpoint, the Kaplan–Meier curve for OS from the initial surgery (initial diagnosis) showed a median OS of 28.8 (20.1–37.5) months (Extended Data Fig. 2a).

Because the action of G47∆ does not theoretically depend on the genetic background of tumor cells, and testing for isocitrate dehydrogenase 1 (IDH1) mutation was not regularly performed in 2014 when this trial protocol was submitted to the Japanese Pharmaceuticals and Medical Devices Agency (PMDA), the status of IDH1 mutation was not included as a covariate of this study. However, all 19 patients were examined post hoc with regard to the IDH1 status, and IDH1 mutation was found in 6 of 19 patients (Table 1). Kaplan–Meier analysis showed that median OS after G47Δ initiation was not affected by IDH1 status (wild type, 20.9 (13.6–28.2) months; mutant type, 19.4 (17.4–21.4) months; *P* = 0.899, log-rank test) (Fig. 1c). Kaplan–Meier analysis also showed that median OS from the initial surgery was not affected by IDH1 status (wild type, 28.8 (17.6–40.0) months; mutant type, 23.6 (16.1–31.1) months; *P* = 0.769, log-rank test; Extended Data Fig. 2b).

**Extended Data Fig. 3:**
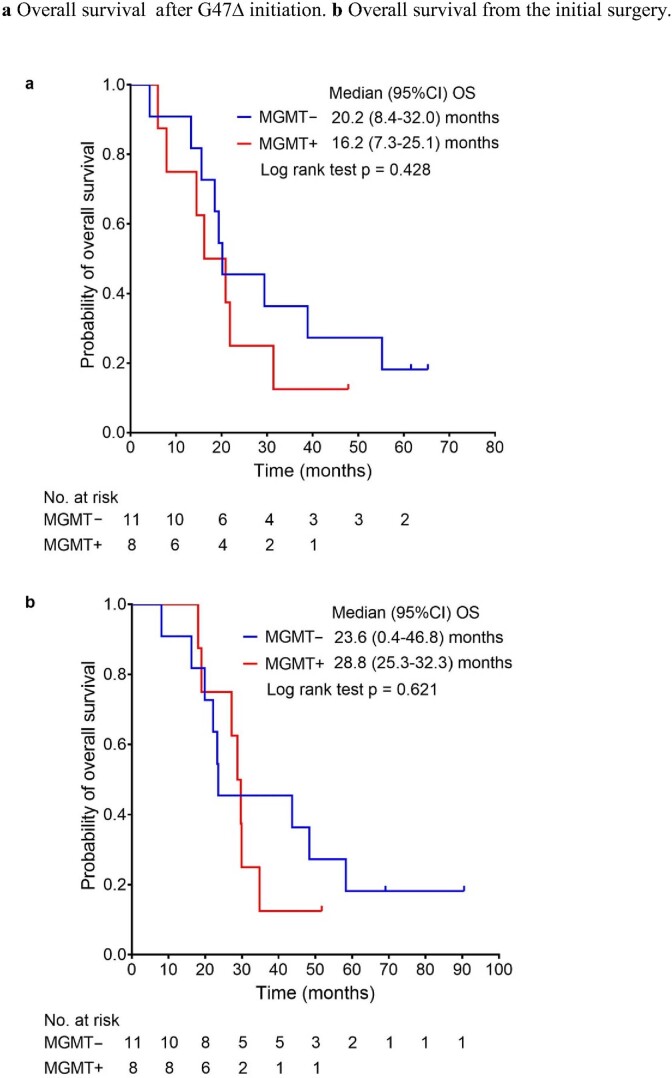
Kaplan-Meier curves based on MGMT expression. **a** Overall survival after G47Δ initiation. **b** Overall survival from the initial surgery. Source data

MGMT methylation status was available for five patients (26.3%) from referring hospitals; three were unmethylated and two were methylated (Table 1). The paraffin-embedded slide sections of initial surgery provided from referring hospitals and the biopsy specimens from this trial were not enough to extract sufficient amounts of DNA to perform methylation-specific PCR. Alternatively, we performed immunohistochemistry for the expression of MGMT using the provided paraffin-embedded slide sections post hoc. MGMT immunohistochemistry was negative (−) in 11 of 19 patients (57.9%), positive (+) in 8 of 19 (42.1%) and strongly positive (++) in none (Table 1). Kaplan–Meier analyses showed that median OS was not affected by MGMT expression, both after G47Δ initiation (MGMT−, 20.2 (8.4–32.0) months; MGMT+, 16.2 (7.3–25.1) months; *P* = 0.428, log-rank test) and from the initial surgery (MGMT−, 23.6 (0.4–46.8) months; MGMT+, 28.8 (25.3–32.3) months; *P* = 0.621, log-rank test) (Extended Data Fig. 3).

As one of the secondary endpoints, the best overall response between the first G47∆ administration and 24 months after the last administration was PR in 1 patient (5.3%) and stable disease in 18 patients (94.7%). The discrepancy between the survival benefit and the tumor response assessed on MRI is likely characteristic to G47∆ treatment. The low response rate was as expected, because the target lesion typically enlarges after G47∆ administration and maintains the size for a certain duration as described below.

**Extended Data Fig. 4:**
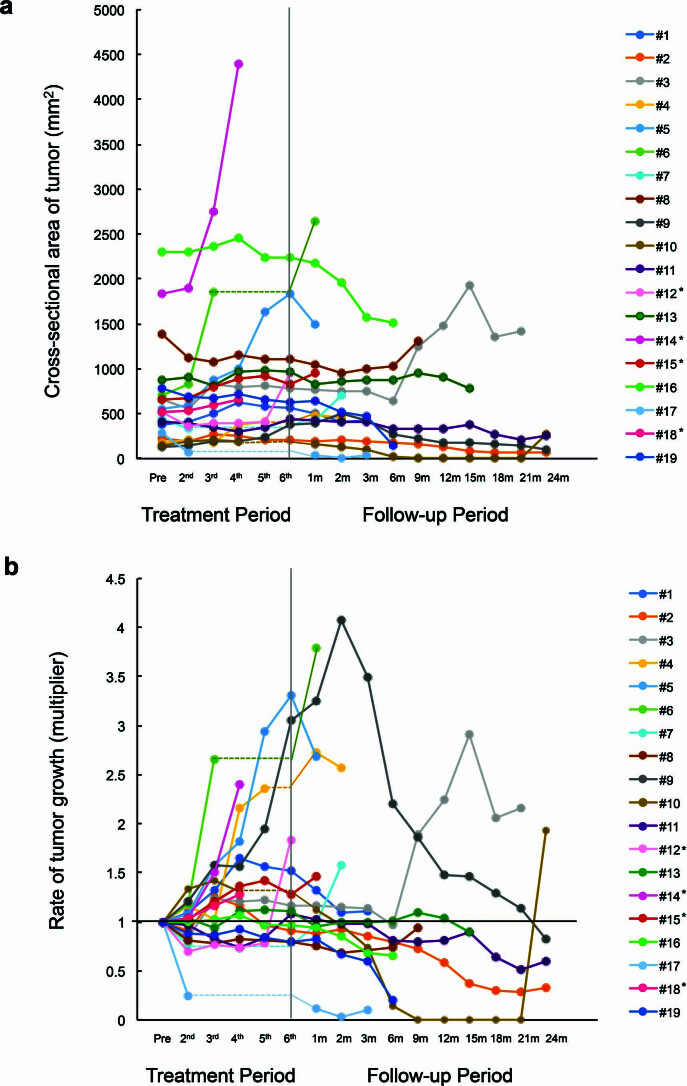
Time course of cross-sectional area of the target lesion of individual patients. **a** Cross-sectional area (CSA) of the target lesion expressed as actual area at every assessment point. The median CSA of the tumor at baseline was 519.9 mm^2^ (range, 122.4-2,306.7 mm^2^). **b** CSA of the target lesion expressed as a ratio relative to the pre-treatment area. The target lesions tended to show a temporary increase in area during G47∆ treatment. Twelve patients (63.2%) showed a decrease in target lesion area for a certain time compared to pre-treatment. In patients who did not receive G47∆ for the maximum of 6 times, the period during which they were not administered is expressed as a horizontal dotted line. * indicates patients who received corticosteroids. Source data

The time course of cross-sectional area of the target lesion of each patient on MRI without bevacizumab treatment is shown in Extended Data Fig. 4. The reduction in target lesion areas observed in some patients at later time points was not associated with corticosteroid administration. Three patients (no. 6, no. 14, no. 18) died within 1 yr of G47∆ therapy (<1-yr group), whereas five patients (no. 2, no. 9, no. 10, no. 11, no. 17) survived longer than 3 yr after G47∆ therapy (>3-yr group; Table 2). The mean tumor area (±s.d.) at the initiation of G47∆ therapy of the <1-yr group and that of the >3-yr group were 1,014.1 ± 714.4 mm^2^ and 238.3 ± 120.0 mm^2^, respectively, indicating that patients who survived >3 yr had significantly smaller initial tumor sizes than those who survived <1 yr (*P* = 0.036, Mann–Whitney *U*-test).

### Safety

Safety was one of the secondary endpoints. For the total study population, 19 patients (100.0%) experienced G47∆-related adverse events (Table 3). The major G47∆-related adverse events included fever (89.5%), vomiting (57.9%), nausea (52.6%), lymphocyte count decrease (47.4%) and white blood cell decrease (31.6%). The G47∆-related grade ≥3 adverse event of lymphocyte count decrease occurred in five patients (26.3%), but all recovered without any treatment. The only serious adverse event attributable to G47∆ was fever (grade 2) in one patient (5.3%) that caused a prolongation of hospitalization. Overall adverse events are summarized in Supplementary Table 1. A grade 5 event occurred in one patient who died in the bath 15 months after the last G47∆ administration. Autopsy revealed that the lesion was well controlled and the death ‘not related’ to G47Δ.

**Table 3:** Treatment-emergent adverse events attributable to G47Δ according to severity grade, *n* = 19

Body system and adverse event, *n* (%)	Grade 1	Grade 2	Grade 3	Grade 4	Grade 5
Patient number with any adverse event	2 (10.5)	10 (52.6)	5 (26.3)	2 (10.5)	
General disorder					
Fever	6 (31.6)	10 (52.6)	1 (5.3)	0 (0)	0 (0)
Gastrointestinal disorder					
Vomiting	3 (15.8)	7 (36.8)	1 (5.3)	0 (0)	0 (0)
Nausea	5 (26.3)	5 (26.3)	0 (0)	0 (0)	0 (0)
Nervous system disorder					
Seizure	0 (0)	3 (15.8)	0 (0)	0 (0)	0 (0)
Cerebral edema	2 (10.5)	1 (5.3)	0 (0)	0 (0)	0 (0)
Neuropathy–sensory	1 (5.3)	0 (0)	0 (0)	0 (0)	0 (0)
Headache	1 (5.3)	0 (0)	0 (0)	0 (0)	0 (0)
Blood					
Anemia	1 (5.3)	0 (0)	0 (0)	0 (0)	0 (0)
Investigations					
Lymphocyte count decreased	1 (5.3)	3 (15.8)	3 (15.8)	2 (10.5)	0 (0)
White blood cell count decreased	2 (10.5)	3 (15.8)	1 (5.3)	0 (0)	0 (0)
Neutrophil count decreased	0 (0)	2 (10.5)	1 (5.3)	0 (0)	0 (0)
Platelet count decreased	3 (15.8)	0 (0)	0 (0)	0 (0)	0 (0)
Bilirubin increased	1 (5.3)	0 (0)	0 (0)	0 (0)	0 (0)
γ-Glutamyl transpeptidase increased	1 (5.3)	0 (0)	0 (0)	0 (0)	0 (0)
PT-INR increased	1 (5.3)	0 (0)	0 (0)	0 (0)	0 (0)
Hypoalbuminemia	1 (5.3)	0 (0)	0 (0)	0 (0)	0 (0)
Hyponatremia	1 (5.3)	0 (0)	0 (0)	0 (0)	0 (0)

CTCAE v.4.03 Japanese version was used to determine each grade. PT-INR, prothrombin time-international normalized ratio.

### Viral shedding

The viral shedding study was an exploratory endpoint. Blood, urine and saliva samples were collected at designated time points as described in the Methods. G47Δ DNA was detected from the blood of patient no. 4 on day 0 only. All other samples were negative by quantitative PCR.

### Imaging

**Fig. 2:**
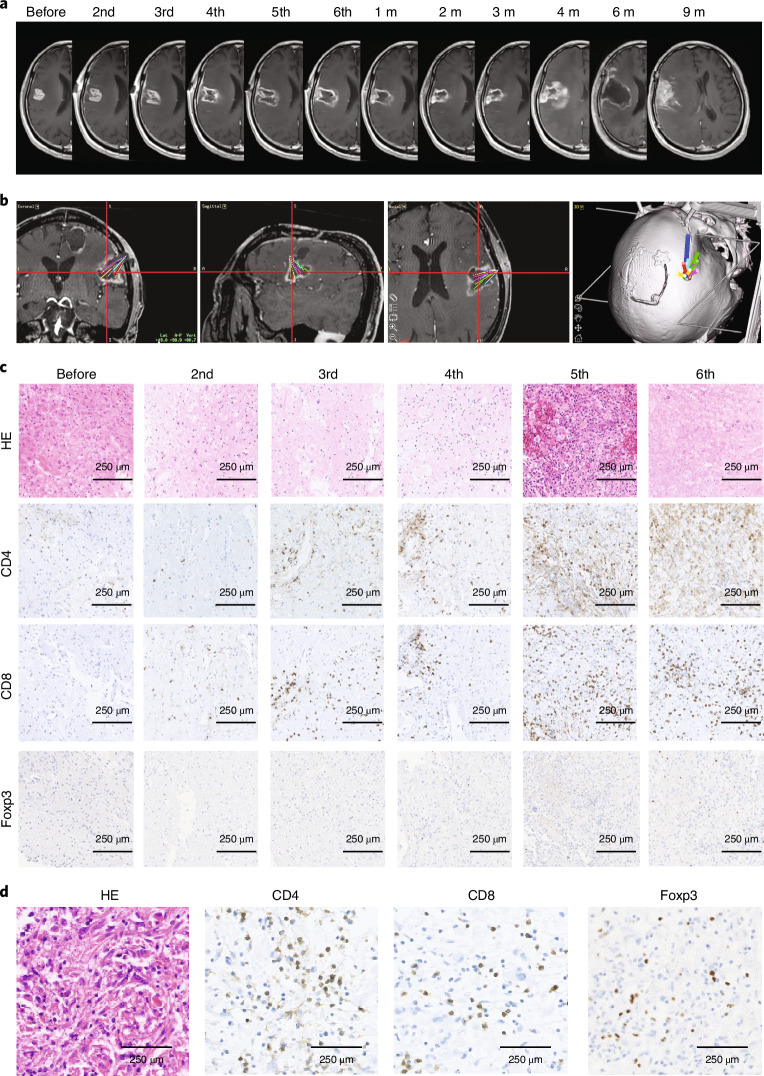
Representative case (patient no. 1) treated with G47Δ. **a**, MRI images at indicated observation time points. Characteristic MRI changes were observed at every G47Δ administration, that is, a clearing of contrast-enhancement at the injection site and an enlargement of the entire target lesion. G47∆ was injected to different coordinates from previous injections, so the area of contrast-enhancement clearing increased as the doses increased, leading to a large hollow within the target lesion with an increase in diameter after six G47∆ doses. The MRI changes ceased after the last G47∆ injection and the target lesion stayed stable until 4 months after G47∆ therapy, when the tumor showed a regrowth. The regrown tumor was resected at 6 months but further regrew at 9 months, and the patient died of exacerbation of the disease at 16.2 months after G47∆ initiation. **b**, Planning MRI using StealthStation Surgical Navigation System at the 6th dose, displaying administration routes from the 2nd to 6th dose (10 injections) overlaid in the same image. **c**, Histology of biopsy specimens. Biopsies were performed before indicated injections and were obtained from coordinates different from previous G47∆ injections. CD4^+^ and CD8^+^ lymphocytes infiltrating within the tumor increased abundantly in number as G47∆ injections were repeated. In contrast, the number of tumor-infiltrating Foxp3^+^ cells remained low throughout repeated G47∆ injections. Representative of four biopsy specimens. **d**, Histology of resected tumor at regrowth. The numbers of tumor-infiltrating CD4^+^ and CD8^+^ lymphocytes remained high in the regrown tumor resected 6 months after the last G47∆ administration. A higher number of Foxp3^+^ cells are observed in the tumor at regrowth than in tumors during G47∆ treatment. Representative of three tissue samples. HE, haematoxylin and eosin; m, month(s).

**Extended Data Fig. 5:**
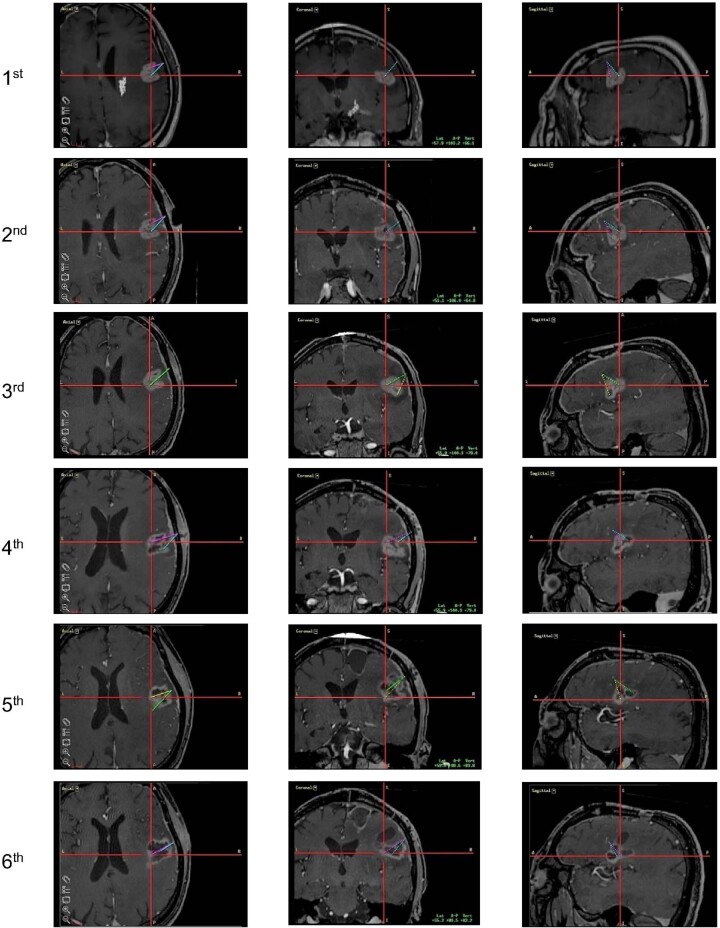
Planning MRI of a representative case (patient no. 1). Individual planning MRI of patient no. 1 using StealthStation Surgical Navigation System from the 1st to the 6th dose displaying 2 administration routes for each dose. Axial (left), coronal (middle) and sagittal (right) sections are shown for each dose. G47∆ was injected into viable tumor sites, depicted as contrast-enhanced portions on MRI, remaining from previous injections.

MRI analyses were an exploratory endpoint. Two MRI features commonly observed in all patients in the FIH trial were also observed in this phase 2 trial: (1) clearing of contrast-enhancement at the injection site; and (2) mild enlargement of target lesions (Fig. 2a). This was observed immediately after G47Δ administration and occurred repeatedly after every G47∆ administration for up to six doses. As G47∆ was administered to a different coordinate for each injection and at two sites within the tumor for each dose (Fig. 2b and Extended Data Fig. 5), the area of clearing typically increased as the doses increased, leading to a large hollow within the target lesion with an increase in diameter after repeated G47∆ doses, mimicking aerial bombing of a surrounded field (Fig. 2a). These MRI changes generally ceased once G47∆ administration was terminated, and the target lesion stayed stable until tumor regrowth (Fig. 2a).

**Fig. 3:**
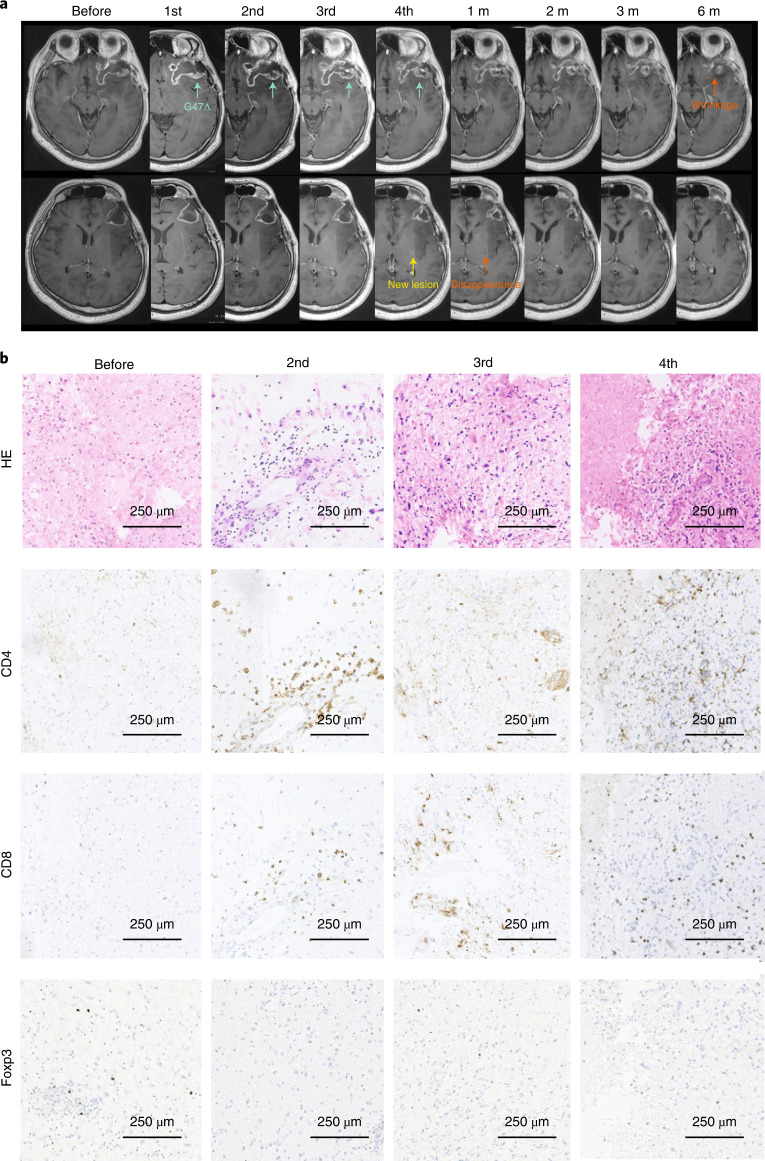
Representative case (patient no. 10) showing a long-term efficacy via antitumor immunity. **a**, MRI images at indicated observation time points. G47∆ was injected into the target lesion (green arrows), causing the characteristic appearance of contrast-enhancement clearing at the injection site and an enlargement of the entire target lesion. After four doses, a new lesion (yellow arrow) appeared in the left basal ganglia remote from the target lesion, so G47∆ therapy was terminated. However, at the first follow-up 1 month after the last G47∆ administration, the new lesion disappeared (orange arrow at 1 month). Eventually, in the observations that followed, the target lesion decreased in size (orange arrow at 6 months). Remote new lesions further appeared at 24 months and the patient died of exacerbation of the disease at 38.9 months after G47∆ initiation. **b**, Histology of biopsy specimens. Biopsies were performed before indicated injections and were obtained from coordinates different from previous G47∆ injections. Similar to patient no. 1, CD4^+^ and CD8^+^ lymphocytes infiltrating within the tumor increased abundantly in number as G47∆ injections were repeated, whereas the number of tumor-infiltrating Foxp3^+^ cells remained low throughout repeated G47∆ injections.

**Extended Data Fig. 6:**
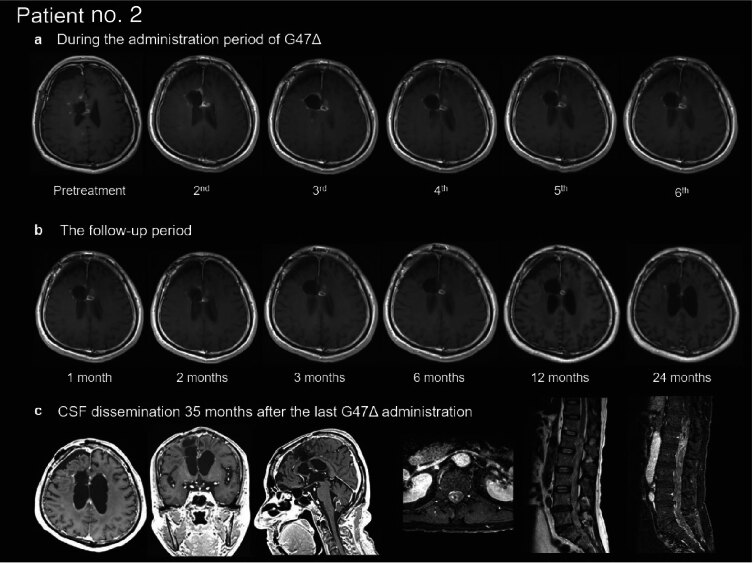
Representative case (patient no. 2) showing cerebrospinal fluid dissemination at progression. **a** Characteristic MRI changes are observed with an overall lesion size increased during the administration period. **b** Shrinkage of the target lesion is observed at 12 months after the last G47Δ administration. **c** Cerebrospinal fluid (CSF) dissemination is observed in the brain and in the spinal cord at 35 months after the last G47Δ administration. Note that the target lesion is well controlled. The disease course since the initiation of G47Δ therapy was 55.2 months.

**Extended Data Fig. 7:**
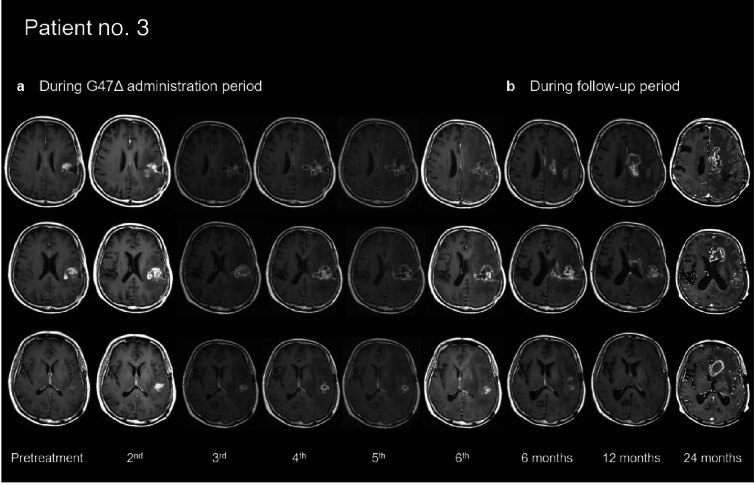
Representative case (patient no. 3) showing target lesion shrinkage and subsequent progression adjacent to but away from G47∆ injected sites. **a** Characteristic MRI changes are observed, resulting in a large clearing area within, and an enlargement of, the target lesion. **b** The target lesion is reduced in size at 6 months after the last G47Δ administration. At 12 months, while the target lesion keeps shrinking, a progression is seen in the brain adjacent to the target lesion anterior-medially. The progression continues at 24 months. The disease course since the initiation of G47Δ therapy was 31.4 months.

We also observed that target lesions were relatively well controlled after G47∆ therapy. However, at progression, we often observed remote new lesions (Fig. 3a), intrathecal dissemination (Extended Data Fig. 6) and tumor extension to sites adjacent to but away from the target lesion (Extended Data Fig. 7).

Long-term efficacy, supposedly via antitumor immunity, was observed in patient no. 10 (Fig. 3). This patient was judged as having progressive disease due to the appearance of a remote new lesion, so G47∆ therapy was terminated after four administrations. However, the new lesion disappeared after 1 month, followed by eventual shrinkage of the target lesion.

### Histology

**Extended Data Fig. 8:**
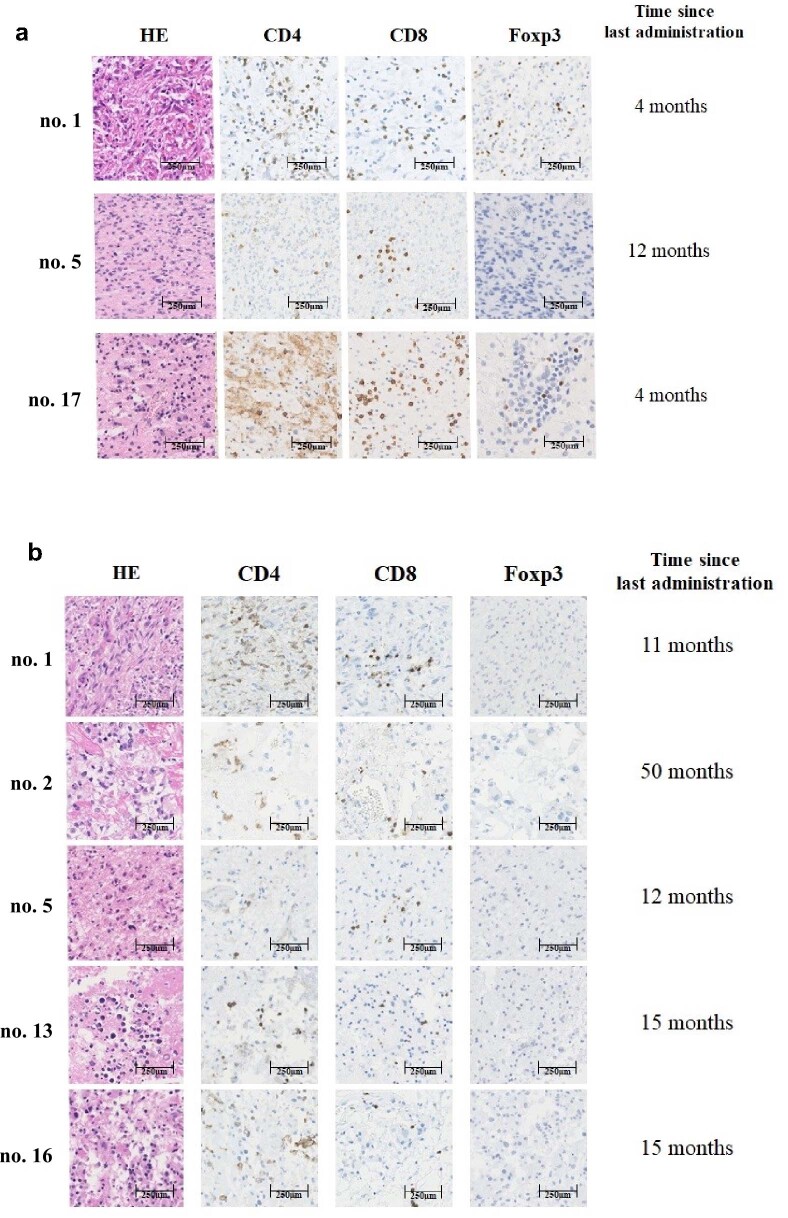
Histology of regrown tumors at reoperation and brain lesions at autopsy. **a** After G47∆ therapy, 3 patients underwent craniotomy for tumor resection of regrown tumors (no. 1 and no. 17 at 4 months and no. 5 at 12 months after the last G47∆ administration). Infiltrating numbers of CD4 + and CD8 + lymphocytes remained abundantly increased in the two cases at 4 months and to some extent in the case at 12 months. Increased numbers of Foxp3+ cells were found in the two cases at 4 months and both underwent reoperation, but not found in the case at 12 months. Each is representative of 3 tissue samples. **b** Among 5 patients who underwent autopsy, 4 patients died of tumor progression (no. 1, no. 2, no. 5 and no. 16) and an infiltration of CD4+ and CD8+ lymphocytes and a low number of Foxp3+ cells persisted at autopsy 11 to 50 months after the last G47Δ administration. In patient no. 13 whose target lesion was well controlled at the time of death, necrosis and calcification, but no viable tumor cells, were found in the brain at autopsy 15 months after the last G47∆ administration. Each is representative of 3 tissue samples.

The histology study was an exploratory endpoint. Biopsies were performed immediately before each G47∆ injection. All patients were confirmed to have viable glioblastoma in at least one of the biopsies. Biopsies also confirmed that all recurrent cases were not pseudoprogression. As G47∆ was injected to different coordinates, biopsy results reflected the histopathology distant from previous G47∆ injections. The histology was characterized by: (1) infiltration of CD4^+^ and CD8^+^ lymphocytes within the tumor that increased abundantly in number as G47∆ injections were repeated; and (2) a low number of Foxp3^+^ cells that remained so despite repeated G47∆ injections (Figs. 2c and 3b and Supplementary Fig. 1). In one patient who showed tumor regrowth 4 months after G47∆ therapy, the high numbers of CD4^+^ and CD8^+^ lymphocytes persisted whereas the number of Foxp3^+^ cells increased (Fig. 2d). Histology results of regrown tumors at reoperation and brain lesions at autopsy are described in Extended Data Fig. 8.

### Treatment after G47∆

Of 15 patients who experienced disease progression after G47Δ, 9 patients received bevacizumab every 4 weeks, 3 patients received bevacizumab after reoperation and 4 patients received extended field stereotactic radiotherapy (Table 2)^7^. One patient with a temozolomide allergy received nimustine as second-line therapy. Sixteen patients received steroids (dexamethasone at mean dose of 13.2 mg ×6 d) after symptomatic progression. At the time of writing, 16 of 19 patients had died with 5 patients having undergone autopsy.

## Discussion

This trial demonstrates the efficacy and safety of G47Δ for residual or recurrent glioblastoma. The 1-yr survival rate of 84.2% and the median OS and PFS of 20.2 months and 4.7 months, respectively, after G47Δ initiation compare favorably with other treatments. Pooled data from 16 trials of various chemotherapy agents for treating recurrent glioblastoma reported a median OS of 5.0 months and a median PFS of 1.8 months (ref. ^8^). Median OS of 6.6 months and 5.3 months are reported for temozolomide monotherapy rechallenge in patients with recurrent glioblastoma receiving adjuvant temozolomide and after a temozolomide-free interval, respectively^9^. A phase 2 study using bevacizumab reported a median OS of 9.2 months in recurrent glioblastoma^10^. Immune checkpoint inhibitors and various molecule-targeted therapies have not shown improved efficacy for recurrent glioblastoma, with median OS of 4.4–9.9 months (refs. ^11,12^). In patients with newly diagnosed glioblastoma, a randomized trial showed a median OS of 20.9 months for the TTF–temozolomide group versus 16.0 months in the temozolomide-alone group^1^. However, in patients with recurrent glioblastoma, TTF failed to provide significant survival advantage over chemotherapy (median OS 6.6 versus 6.0 months)^13^. In this G47∆ phase 2 trial, the median OS was 28.8 months from the initial surgery.

Since the status of IDH1 mutation was not included in the eligibility criteria, it was examined as a post hoc study, and 6 of 19 patients were found to be IDH1 mutated. However, the difference in IDH1 mutation was shown to have no impact on the OS both after G47∆ initiation and from the initial surgery in this study. It has been reported that there is no difference in survival time after recurrence of glioblastoma with or without IDH mutation^14^. Six patients had carmustine (BCNU) wafers (Gliadel, Eisai, for Arbor Pharmaceuticals) placed in the tumor cavity at their initial surgeries, and it happened that five of six were IDH1 mutated. Although BCNU wafers (Gliadel) were reported effective for newly diagnosed malignant glioma, a survival benefit was not shown in the subset of patients with glioblastoma in a prospective, open-label, randomized trial^15^, and no significant survival benefit was found for glioblastoma even in the long-term follow-up of this trial^16^. Rather, it has been reported that the toxicity after Gliadel use is significantly higher, for patients with both newly diagnosed and recurrent glioblastoma^17^. The definition of glioblastoma was changed in the 2021 World Health Organization (WHO) classification, and glioblastoma in this study is a mixture of glioblastoma and grade 4 IDH-mutant astrocytoma according to the new WHO classification^18^.

The survival benefit without a remarkable tumor response on MRI is likely characteristic of G47∆ therapy. The overall response rate was only 5.3%, with one PR case. Lymphocytes, presumably infiltrating towards tumor cells, accumulate within the tumor with repeated G47∆ doses, so the actual antitumor effects are not reflected on image studies. This is quite opposite to the situation with bevacizumab, which shows excellent rates of radiographic response, but tumors continue to progress without survival benefits^19^. The presence of many cases that maintained stable disease in this phase 2 study was one of the factors of the drug approval by the PMDA.

Regarding the dosing schedule, G47∆ was administered repeatedly for a maximum of six times. A preclinical study with G207 demonstrated that the efficacy of six intratumoral injections was superior to the efficacy of a single injection with a tenfold higher dose^20^. The expected survival period of a patient with glioblastoma after recurrence is about 6 months with standard chemotherapy^8^. Furthermore, from an experienced neurosurgeon’s perspective, repeating a burr hole surgery six times could be considered feasible without causing patients excessive suffering. Six doses are also acceptable cost wise for patients in Japan, because national health insurance covers the treatment during clinical trials and after drug approval, including surgery and in-patient care.

Adverse events related to G47∆ were mostly restricted to those caused by immune responses and are likely consequences of the immune system attempting to eliminate an unnaturally large load of virus that robustly replicates in a localized area. As systemic responses, fever and headache were frequently manifested and, as a local response, tumor swelling was commonly observed. Fever and vomiting were the only two grade 3 adverse events attributable to G47∆ and unrelated to blood cell counts. A similar adverse event profile, both systemic and local, has been noted with vaccines, such as the Pfizer-BioNTech COVID-19 vaccine^21^. Decreased lymphocyte count may be virus-related, but could not be distinguished from an adverse event caused by temozolomide^22^. Rationally, an effective countermeasure against immune responses is the use of corticosteroids^23^. In the FIH study, we found that corticosteroid administration immediately diminished the immune response-related adverse events including fever and tumor swelling, and did not interfere with long-term antitumor immunity development when the duration was kept within 1 week (ref. ^5^). In this phase 2 study, corticosteroids were used in four patients (21.1%) to suppress G47∆ adverse events, only once during the entire treatment in all cases. In contrast, a recombinant nonpathogenic polio-rhinovirus chimera (PVSRIPO) reportedly caused severe brain edema and neurologic symptoms related to peritumoral inflammation when injected intratumorally in patients with glioblastoma, even causing death in one patient, and all patients required corticosteroid administration^24^.

Dose-limiting toxicity has not been observed with G47∆. The concept of maximum tolerated dose being equivalent to the optimal dose in the development of chemotherapy drugs may not apply to oncolytic virus development. In fact, dose-limiting toxicity has not been observed with G207, the parental virus of G47∆, and the highest dose tested in the first phase 1 study was 3 × 10^9^ plaque-forming units (p.f.u.) per dose^25^, but lower doses, and not this highest dose, were used in the subsequent clinical trials^26,27^. In practice, there are limits to the amount of virus per ml that can be manufactured, depending on the type of virus, and, in general, the higher the concentration, the higher the production cost when mass-produced under good manufacturing practices. Therefore, for practical realization of oncolytic virus therapy, it is also necessary to consider that the amount of oncolytic virus used to show efficacy can be manufactured as a product at a reasonable cost so that the price is affordable for patients after marketing.

At the time of this manuscript submission, three patients were alive and stable for more than 3 yr after the last G47∆ administration. A proportion of G47∆-treated patients experienced long-term survival in this phase 2 study as well as in the FIH trial^5^. Such a proportion of long-term survival was similarly observed in the phase 1 trial of PVSRIPO in patients with glioblastoma^24^. From in vivo replication studies in animals and biopsy specimens obtained from the exact coordinates of the first G47∆ injection in the FIH trial^5^, we estimate that G47∆ injected into glioblastoma is eliminated by the immune system within 4 weeks (refs. ^2,28^). Target lesions were generally well controlled. These facts indicate that a delayed effect of G47∆ via induction of systemic antitumor immunity may be the major mechanism for long-term disease control.

To support the notion that specific antitumor immune responses are boosted after multiple G47∆ injections, the biopsy histology revealed that tumor-infiltrating CD4^+^ and CD8^+^ lymphocyte populations increase with repeated G47∆ injections. These lymphocytes which recognize and infiltrate towards tumor cells appear almost immediately after G47∆ therapy. However, it takes approximately 4 months or more after initiation of G47Δ therapy until the antitumor immunity causes tumor shrinkage, although it may be acting from much earlier to suppress tumor growth. Therefore, lymphocytes that rapidly infiltrate the tumor may recognize the tumor cells but their exact functions are yet to be elucidated. Tumor-infiltrating CD4^+^ and CD8^+^ lymphocytes persisted for a long time (>50 months), as observed in resected regrown tumors and at autopsies. Cells with Foxp3, which acts as a master transcription factor for regulatory T cells^29^, were rarely found in biopsy specimens despite repeated G47∆ injections. Since an increased number of Foxp3^+^ cells were observed in some of the tumors that regrew after G47∆ therapy, inhibition of Foxp3^+^ cells may be relevant to G47∆ efficacy. We also observed that lesions close to G47∆ injection sites were relatively well controlled, although tumor regrowth was often observed remote from the target lesion or as dissemination via cerebrospinal fluid. Interestingly, in a phase 3 trial of the oncolytic HSV-1 talimogene laherparepvec (T-Vec), patients with stage IIIB–IVM1c melanoma also experienced more frequent immune-related antitumor effects on nontreated lesions close to treated lesions than distant ones^30^. The common observation with G47∆ in the brain and T-Vec outside the brain, that nontreated lesions close to the treated lesion are better controlled than distant ones, suggests the presence of action mechanisms of antitumor immunity that are not yet known.

The characteristic MRI changes were observed after every G47∆ injection regardless of repeated administration. The biopsy histology of the FIH trial revealed that clearing at the injection site reflected the area of virus replication and tumor cell destruction^5^. This study further demonstrates that the immediate enlargement of the target lesion is caused by rapid infiltration of lymphocytes within the tumor, which may be called ‘immunoprogression’ and should be clearly distinguished from pseudoprogression that occurs after radiation therapy. Although it has been reported that angiogenesis is stimulated by G47Δ in a mouse model^31^, the enlargement of the target lesion occurs immediately and every time after G47∆ injection, so rapid lymphocyte infiltration is more likely the contributing mechanism than angiogenesis.

The response criteria adopted in this trial functioned well, and 12 patients (63.2%) were able to receive a total of 6 doses without being prematurely judged to have tumor progression. Of these 12 patients, 7 patients showed a reduction in target lesion size after a temporary increase, typically 9–12 months after the last G47Δ dose. Since G47∆ replication supposedly ceases much earlier, such shrinkage of the target lesion is likely due to a delayed effect of G47∆ via elicited antitumor immunity. In addition, patients that survived >3 yr after G47∆ therapy had smaller initial tumor sizes than those that survived <1 yr. Thus, G47Δ treatment earlier in the course of this disease may be warranted to achieve high efficacy and potentially obtain a cure. One patient from this trial and one patient from the FIH trial showed long-term survival, with lesions controlled after only two G47∆ administrations, so the number of administrations required for an effective response apparently varies among individuals. An adequate frequency of G47∆ injections is expected to become clear in a planned post-marketing investigation.

G47Δ represents an evolved oncolytic virus clinically tested for gliomas that include Newcastle disease virus, reovirus, parvovirus, adenovirus, polio virus and vaccinia virus^32^. PVSRIPO, a recombinant poliovirus-rhinovirus chimera mentioned earlier, has shown efficacy in a single phase 1 clinical trial of 61 patients, although the improvement in median OS was modest^24^. Among adenovirus candidates, DNX-2401 has shown evidence of viral-induced necrosis in patients with malignant glioma and has been fast-tracked as an orphan drug by the US Food and Drug Administration^33^. G207 was recently shown to be safe in children with supratentorial high-grade glioma^34^.

During this phase 2 trial, G47∆ was designated as a SAKIGAKE (breakthrough therapy) Product, and further as an Orphan Regenerative Medicine Product for malignant glioma by the Japanese Ministry of Health, Labor and Welfare (MHLW), allowing fast-track review and approval. Hence, this phase 2 trial served as a pivotal study, and led to the conditional and time-limited approval of G47∆ for malignant glioma by the MHLW on 11 June 2021 as a Gene Therapy Product, the first oncolytic virus drug in Japan (https://www.daiichisankyo.com/files/news/pressrelease/pdf/202106/20210611_E_47.pdf; DELYTACT oncolytic virus G47∆ approved in Japan for treatment of patients with malignant glioma). The study population was rather small, with 97 surgeries in 19 patients, one factor for which was that this was an academia-initiated drug development for rare cancer in the Japanese medical system under Japanese regulations for gene therapy. It is planned that all patients using commercially distributed G47∆ be registered and followed, and clinical data evaluated against a control population of patients under PMDA supervision in the next 7 yr. G47Δ is perhaps the first new drug since temozolomide and the first new treatment since TTF that shows a survival benefit for glioblastoma, and provides a potential cure in a proportion of patients. Now that this nonconventional therapeutic modality is approved as a treatment option in Japan, the standard care of malignant glioma may change in the future.

To date, G47Δ has been shown to be efficacious via the same mechanism of action in various solid tumors in vivo, including prostate cancer, gastric cancer, hepatocellular carcinoma, tongue cancer, esophageal cancer, breast cancer, neuroblastoma and malignant peripheral nerve sheath tumor^2,3,35–41^. Oncolytic HSV-1, including G47∆, does not infect normal bone marrow-derived cells^2,42^, but recent studies show that even some blood cancers are susceptible to G47∆ (refs. ^43,44^). Further, G47Δ has been shown to have augmented efficacy when used in combination with immune checkpoint inhibitors^38,45,46^. We will pursue expansion of indications for G47Δ to other solid cancers as swiftly as possible.

## Methods

### Study design and participants

This investigator-initiated phase 2 trial was conducted at a single institution (the Institute of Medical Science Hospital, the University of Tokyo (IMSUT Hospital)) in Japan (UMIN-CTR Clinical Trial Registry, UMIN000015995, registered and posted on 18 December 2014). The first patient enrollment date was 19 May 2015 and the last patient enrollment date was 18 April 2018. This study was approved by the PMDA on 29 August 2014. Under the guidance of the PMDA, it was recommended that this study be conducted at a single institution for safety reasons, as this was the first PMDA-supervised clinical trial of oncolytic virus therapy. The data cut-off date for the Case Study Report submitted to the PMDA was 6 April 2020, although survival-related data continued to be collected until 1 March 2022 and are presented here.

The full inclusion and exclusion criteria are listed below.

Inclusion criteria:

(i) age 18 yr or older
(ii) a pathologically confirmed diagnosis of glioblastoma with a persistent or recurrent tumor (represented as a contrast-enhanced lesion of ≥1.0 cm on MRI) despite having received radiation therapy and temozolomide
(iii) a Karnofsky Performance Scale score ≥60%
(iv) a constant steroid dosage within 1 week on the day of eligibility assessment
(v) able to use barrier-type contraceptive during the study and for 6 months after the last dose of drug
(vi) potential to survive ≥3 months
(vii) laboratory data meet the following criteria (white blood cell count > 2,000 per mm^3^, neutrophil count > 1,000 per mm^3^, platelet count > 100,000 per mm^3^, haemoglobin level > 9.0 g dl^−1^, prothrombin time-international normalized ratio (PT-INR) ≤ 1.3 times the upper limit of the institution baseline, serum creatinine < 1.7 mg dl^−1^, aspartate aminotransferase (AST) and alanine aminotransferase (ALT) ≤ 4 times the upper limit of the institution baseline, total bilirubin or direct bilirubin ≤ 1.5 mg dl^−1^).

Exclusion criteria:

(i) confirmed presence of any of the following tumors: metastatic tumors outside of the brain; multiple intracranial malignant glioma lesions (considered as multiple contrast-enhanced lesions with separate Fluid Attenuated Inversion Recovery (FLAIR) high-intensity areas); tumors located in the ventricles, brainstem or posterior fossa; or tumors for which the investigational product (G47∆) must be administered via the ventricles, subependymal or subarachnoidal dissemination
(ii) past or current medical history of any of the following: encephalitis, multiple sclerosis or other central nervous system infections; positive for human immunodeficiency virus; or conditions in which the use of MRI contrast media is contraindicated (for example, patients with a pacemaker or continuous infusion pump in the body or patients allergic to MRI contrast media)
(iii) any of the following complications: active herpes virus infection; herpes virus infections requiring treatment with an antiviral (acyclovir or valacyclovir); active, poorly controlled infection that prevents surgery; uncontrolled or severe heart failure; diabetes mellitus; hypertension; interstitial pneumonia; renal failure; autoimmune disease; addiction to alcohol or other drugs; other active malignancies
(iv) history of allergy to antivirals (acyclovir or valacyclovir)
(v) history of receiving any of the following treatments or operations: other investigational products or investigational therapies (within 30 d before G47∆ administration, or within 5 times the half-life of other investigational products or investigational therapies, whichever is longer); brain tumor resection (within 30 d before G47∆ administration); gene therapy or oncolytic virus therapy other than G47∆; bevacizumab (within 30 d before G47∆ administration)
(vi) pregnancy or lactation
(vii) otherwise considered ineligible for inclusion by the principal investigator or the subinvestigator

In the above exclusion criteria, multiple contrast-enhanced lesions with separate Fluid Attenuated Inversion Recovery (FLAIR) high-intensity areas were considered as multiple intracranial malignant glioma lesions.

All patients enrolled in the trial provided written, informed consent. The protocol was approved by the institutional review board of the Institution of Medical Science, the University of Tokyo. Patients were not compensated for trial participation.

Because of the unprecedented administration route of this anti-cancer drug, an intratumoral injection via stereotactic surgery, it was discussed and agreed with the PMDA to target a glioblastoma lesion that was either residual or recurrent, since such was likely to be the disease indication upon drug approval. To minimize a one-institution study bias, all consecutive patients that met the criteria were included in the order registered without exception. All histology specimens used for diagnosis of enrolled patients were reviewed by a neuropathologist on our team to confirm the diagnosis of glioblastoma.

MGMT methylation status was provided from referring hospitals for five patients. IDH1 mutation status and MGMT expression were tested post hoc by immunohistochemistry using paraffin-embedded formalin-fixed slide sections of the initial surgery provided by referring hospitals. Primary antibodies for IDH1 mutation (R132H) and MGMT were anti-IDH1 R132H/DIA-H09 Mouse monoclonal anti-brain tumor marker Clone H09 (Dianova; DIA-H09) and anti-MGMT antibody [MT3.1] (Abcam; ab39253), respectively.

### G47∆ formulation

Test products were prepared at the Therapeutic Vectors Development Center, IMSUT Hospital, and consisted of G47Δ suspended in 10% glycerol/phosphate buffer solution. Formulations were dispensed and stored in polypropylene cryotubes at −80 °C. Before surgery, G47∆ was adjusted to a concentration of 1 × 10^9^ p.f.u. ml^−1^ and kept on ice until use. The titer of the original stock used in this trial was 1.2 × 10^10^ p.f.u. ml^−1^. The clinical lot was tested for stability by titration at sequential time points, which showed that the virus titer of the product was stable for at least 7 d at room temperature and for approximately 2 months at 4 °C. The time from thawing to injection ranged from 1 to 3 h approximately.

### Intervention

G47Δ was administered intratumorally by MRI-guided stereotactic surgery at intervals of 5–14 d for the first and second doses, and up to six doses at intervals of 4 ± 2 weeks for the third and subsequent doses. A total of 1 × 10^9^ p.f.u. per dose in 1 ml of solution was divided equally and injected into 1–3 coordinates within a tumor using the Biopsy/Injection Needle (MES-CG07-200-01, Mizuho). This Biopsy/Injection Needle device was specifically designed for oncolytic virus injection, so that there is little dead space (15 µl) between the attached syringe and the injection needle exit. The device consists of an outer guide (outer cylinder), a biopsy needle and an injection needle, and either of the latter two needles can be inserted into the outer guide without moving the position of the guide. Therefore, the coordinates of biopsy and G47∆ injection were exactly the same. Biopsy always preceded G47∆ injection. G47∆ was injected manually at a speed of 0.2 ml min^−1^. After injection, the injection needle was kept in place for 5 min before retraction to avoid reflux. From the second dose onwards, G47∆ was injected into viable tumor sites, depicted as contrast-enhanced portions on MRI, remaining from previous injections.

Radiation therapy was prohibited during the study. Steroid administration was permitted, but new doses or dose changes had to be recorded. Concomitant temozolomide was allowed, whereas other antineoplastic agents were prohibited.

The criteria for treatment discontinuation were as follows: (1) target lesion size <1 cm; (2) progressive disease judgment under response criteria; (3) aggravation of clinical symptoms; (4) patient’s wish to discontinue treatment or clinical judgment by the principal investigator or subinvestigator.

### Outcomes

The primary endpoint was the 1-yr survival rate after G47∆ treatment initiation. Secondary endpoints included OS and PFS after initial G47Δ administration, tumor response for efficacy and adverse event frequency.

As one of the secondary endpoints, the best overall response during the observation period (2 yr after the last administration) was assessed by consecutive MRI assessments according to the response criteria adopted for this trial. These response criteria were designed for this clinical trial with reference to immune-related response criteria^47^ and approved by the PMDA. MRI data after administration of bevacizumab after disease progression were not used for the overall response rate evaluation.

The response criteria are as follows:

Complete response: two consecutive MRI scans performed at an interval of at least about 4 weeks show complete disappearance of a target lesion, with no appearance of new lesions.

Partial response: two consecutive MRI scans performed at an interval of at least about 4 weeks show a decrease in the sum of areas of target lesions by at least 50% compared with that before the first dose, with no appearance of new lesions.

Stable disease: response other than complete response, partial response or progressive disease.

Progressive disease: two consecutive MRI scans performed at an interval of at least about 4 weeks show an increase in the sum of areas of target lesions by at least 25% compared with that on the respective last MRI scans, or appearance of new lesion(s).

Tumor cross-sectional area was defined as (the largest diameter) × (the diameter perpendicular to the largest diameter) of the circumference of the contract-enhanced lesion on an axial section of MRI.

Adverse events were classified and graded using the National Cancer Institute Common Terminology Criteria for Adverse Events (CTCAE v.4.03) terminology. Differentiation of adverse events caused by G47∆ and those caused by surgical injection procedures was judged by the investigator (neurosurgeon).

### Sample collection for viral shedding

For each patient, blood and urine samples were collected after G47∆ administration on the same day (day 0); blood, urine and saliva samples on days 1, 2 and 3 for the first G47∆ administration; and on days 1, 2, 3 and 7 for the second G47∆ administration onwards for every administration.

### Statistical planning and interim analysis

At planning, this trial assumed achievement of a 1-yr survival rate of 40% based on the results of the FIH trial^5^. Based on the 1-yr survival rate for recurrent glioblastoma after chemo-radiotherapy (14%)^8^, the comparative control value was set to 15%. Assuming a superiority of G47Δ of 5% on one side and a power of 80%, and one interim analysis to be performed, the treatment arm size was calculated as 25 patients. An interim analysis was to be conducted when the number of patients followed for 1 yr from the initiation of study treatment reached 13 patients, so the study was designed to include 30 patients to ensure adequate enrollment. The increase in the number of Type I errors resulting from the interim analysis will be adjusted by the Lan–Demet method using the O’Brien–Fleming type α. The significance level of the hypothesis test will be set at 0.557% on one side. Efficacy and safety analyses included all patients who received at least one dose of G47Δ, and this defined the FAS for efficacy and the safety analysis set for safety.

### Statistical analysis

Patient background factors were aggregated according to the characteristics of the data. For the primary endpoint, a 1-yr survival rate after G47∆ initiation was calculated along with the 95% CI. For the secondary endpoints, an OS after G47∆ initiation, a PFS after G47∆ initiation and an OS from the initial surgery with respective 95% CIs were calculated by the Kaplan–Meier method. Adverse events for the safety assessment were analyzed by event. Data analyses were done with SAS Windows, v.9.4 (SAS Institute), or IBM SPSS Statistics v.22 software (IBM Corporation). For the survival data, 30 d were calculated as 1 month according to the Clinical Study Report submitted to the PMDA.

### Reporting summary

Further information on research design is available in the Nature Research Reporting Summary linked to this article.

## Online content

Any methods, additional references, Nature Research reporting summaries, source data, extended data, supplementary information, acknowledgements, peer review information; details of author contributions and competing interests; and statements of data and code availability are available at 10.1038/s41591-022-01897-x.

## Supplementary information

Supplementary Information Supplementary Table 1 and Fig. 1.

Reporting Summary

## Source data

Source Data Fig. 1 Kaplan–Meier data.

Source Data Extended Data Fig. 2 Kaplan–Meier data.

Source Data Extended Data Fig. 3 Kaplan–Meier data.

Source Data Extended Data Fig. 4 Statistical data (tumor size data over time).

## Data Availability

Any requests for raw and analyzed data will be reviewed by the Institute of Medical Science Hospital, the University of Tokyo. Patient-related data not included in the paper were generated as part of a clinical trial and are subject to patient confidentiality. Any data and materials (for example, tissue samples or imaging data) that can be shared will need approval from the Institute of Medical Science Hospital, the University of Tokyo. Any data shared will be de-identified. Requests should be made to the corresponding author (toudou-nsu@umin.ac.jp); response time will be within approximately 5–10 business days. Source data are provided with this paper.
