# Author Correction: Intratumoral oncolytic herpes virus G47∆ for residual or recurrent glioblastoma: a phase 2 trial

**DOI:** 10.1038/s41591-025-03619-5

**Published:** 2025-03-18

**Authors:** Tomoki Todo, Hirotaka Ito, Yasushi Ino, Hiroshi Ohtsu, Yasunori Ota, Junji Shibahara, Minoru Tanaka

**Affiliations:** 1 Division of Innovative Cancer Therapy, Advanced Clinical Research Center, and Department of Surgical Neuro-Oncology, The Institute of Medical Science, The University of Tokyo, Tokyo, Japan; 2 Department of Data Science, National Center for Global Health and Medicine in Japan, Tokyo, Japan; 3 Leading Center for the Development and Research of Cancer Medicine, Juntendo University, Tokyo, Japan; 4 Department of Pathology, The Institute of Medical Science, The University of Tokyo, Tokyo, Japan; 5 Department of Pathology, Kyorin University School of Medicine, Tokyo, Japan

**Keywords:** CNS cancer, Translational research

Correction to: *Nature Medicine* 10.1038/s41591-022-01897-x, published online 21 July 2022.

In the version of the article initially published, in Fig. 2c, the image for CD4 at the second injection was inadvertently taken from the photograph of CD4 staining at the third injection and now has been replaced with the correct image. The image originally published for CD4 at the second injection was intended to be used for the third injection, as it more closely matches the image from the consecutive section shown for CD8 staining, although the original image for CD4 at the third injection was not incorrect. The image for the third injection for CD4 has therefore been replaced with the one originally published as CD4 staining at the second injection.

For comparison, the original and revised Fig. 2c are available in the Supplementary Information accompanying this amendment. The changes are made in the HTML and PDF versions of the article.

## Supplementary information

Original and revised Fig. 2c

