# Supplementary material for: Intratumoral oncolytic herpes virus G47∆ for residual or recurrent glioblastoma: a phase 2 trial
Source: Nat Med. 2022 Jul 21;28(8):1630–9. doi: 10.1038/s41591-022-01897-x (PMC9388376; doi:10.1038/s41591-022-01897-x)
Supplement: Supplementary file 1 — Supplementary Table 1 and Fig. 1. [file 41591_2022_1897_MOESM1_ESM.pdf]

nature portfolio

https://doi.org/10.1038/s41591-022-01897-x

Supplementary information

Intratumoral oncolytic herpes virus G47Δ for residual or recurrent glioblastoma: a phase 2 trial

In the format provided by the authors and unedited

Supplementary Material for

Intratumoral oncolytic herpes virus G47Δ for residual or recurrent glioblastoma: a phase 2 trial

Tomoki Todo, M.D., Ph.D.[1], Hirotaka Ito, M.D., Ph.D.[1], Yasushi Ino, M.D., Ph.D.[1], Hiroshi Ohtsu, MSc.[2, 3], Yasunori Ota, M.D., Ph.D.[4], Junji Shibahara, M.D., Ph.D.[5], Minoru Tanaka, M.D., Ph.D.[1]

1. *Division of Innovative Cancer Therapy, Advanced Clinical Research Center, and Department of Surgical Neuro-Oncology, The Institute of Medical Science, The University of Tokyo, Tokyo, Japan*
2. *Department of Neurosurgery, The University of Tokyo Hospital, Tokyo, Japan*
3. *Department of Data Science, National Center for Global Health and Medicine in Japan, Tokyo, Japan*
4. *Leading Center for the Development and Research of Cancer Medicine, Juntendo University, Tokyo, Japan.*
5. *Department of Pathology, Kyorin University School of Medicine, Tokyo, Japan*

This pdf file contains:

Table S1. Incidence of adverse events in safety analysis set

Figure S1. Histology of biopsy specimens before each G47Δ administration for each patient (n=19)

Table S1. Incidence of adverse events in safety analysis set

System Organ Class	Grade 1, n (%)	Grade 2, n (%)	Grade 3, n (%)	Grade 4, n (%)	Grade 5, n (%)
Number of patients with adverse events	0 (0.0)	2 (10.5)	9 (47.4)	7 (36.8)	1 (5.3)
General disorders and administration site conditions	3 (15.8)	14 (73.7)	1 (5.3)	0 (0.0)	1 (5.3)
Fever	5 (26.3)	12 (63.2)	1 (5.3)	0 (0.0)	0 (0.0)
Pain	8 (42.1)	0 (0.0)	0 (0.0)	0 (0.0)	0 (0.0)
Peripheral edema	3 (15.8)	0 (0.0)	0 (0.0)	0 (0.0)	0 (0.0)
Gait disturbance	0 (0.0)	2 (10.5)	0 (0.0)	0 (0.0)	0 (0.0)
Poor healing	0 (0.0)	2 (10.5)	0 (0.0)	0 (0.0)	0 (0.0)
Malaise	2 (10.5)	0 (0.0)	0 (0.0)	0 (0.0)	0 (0.0)
Death	0 (0.0)	0 (0.0)	0 (0.0)	0 (0.0)	1 (5.3)
Peripheral swelling	0 (0.0)	1 (5.3)	0 (0.0)	0 (0.0)	0 (0.0)
Nervous system disorder	0 (0.0)	5 (26.3)	12 (63.2)	2 (10.5)	0 (0.0)
Headache	5 (26.3)	8 (42.1)	0 (0.0)	0 (0.0)	0 (0.0)
Cerebral edema	0 (0.0)	7 (36.8)	4 (21.1)	1 (5.3)	0 (0.0)
Decreased level of consciousness	3 (15.8)	3 (15.8)	2 (10.5)	1 (5.3)	0 (0.0)
Convulsive seizure	2 (10.5)	6 (31.6)	1 (5.3)	0 (0.0)	0 (0.0)
Hemiparesis	1 (5.3)	2 (10.5)	5 (26.3)	0 (0.0)	0 (0.0)

Hemiplegia	1 (5.3)	2 (10.5)	4 (21.1)	0 (0.0)	0 (0.0)
Sensory disturbance	3 (15.8)	1 (5.3)	0 (0.0)	0 (0.0)	0 (0.0)
Dysarthria	2 (10.5)	1 (5.3)	0 (0.0)	0 (0.0)	0 (0.0)
Homonymous hemianopsia	0 (0.0)	3 (15.8)	0 (0.0)	0 (0.0)	0 (0.0)
Cerebral infarction	0 (0.0)	1 (5.3)	1 (5.3)	0 (0.0)	0 (0.0)
Mental dysfunction	0 (0.0)	1 (5.3)	1 (5.3)	0 (0.0)	0 (0.0)
Syncope	0 (0.0)	0 (0.0)	2 (10.5)	0 (0.0)	0 (0.0)
Altered state of consciousness	0 (0.0)	0 (0.0)	1 (5.3)	0 (0.0)	0 (0.0)
Aphasia	1 (5.3)	0 (0.0)	0 (0.0)	0 (0.0)	0 (0.0)
Cervicobrachial syndrome	0 (0.0)	1 (5.3)	0 (0.0)	0 (0.0)	0 (0.0)
Dizziness	1 (5.3)	0 (0.0)	0 (0.0)	0 (0.0)	0 (0.0)
Dysesthesia	1 (5.3)	0 (0.0)	0 (0.0)	0 (0.0)	0 (0.0)
Hydrocephalus	0 (0.0)	0 (0.0)	1 (5.3)	0 (0.0)	0 (0.0)
Mydriasis	0 (0.0)	0 (0.0)	0 (0.0)	1 (5.3)	0 (0.0)
Neurological neglect syndrome	1 (5.3)	0 (0.0)	0 (0.0)	0 (0.0)	0 (0.0)
Polyneuropathy associated with malignancy	0 (0.0)	1 (5.3)	0 (0.0)	0 (0.0)	0 (0.0)
Agnosia	0 (0.0)	1 (5.3)	0 (0.0)	0 (0.0)	0 (0.0)
Third cranial nerve disorder	0 (0.0)	0 (0.0)	1 (5.3)	0 (0.0)	0 (0.0)
Thalamic hemorrhage	1 (5.3)	0 (0.0)	0 (0.0)	0 (0.0)	0 (0.0)

Midline brain shift	0 (0.0)	1 (5.3)	0 (0.0)	0 (0.0)	0 (0.0)
Decerebrate posture	0 (0.0)	0 (0.0)	0 (0.0)	1 (5.3)	0 (0.0)
Gastrointestinal disorder	6 (31.6)	10 (52.6)	2 (10.5)	0 (0.0)	0 (0.0)
Nausea	5 (26.3)	9 (47.4)	0 (0.0)	0 (0.0)	0 (0.0)
Vomiting	5 (26.3)	7 (36.8)	1 (5.3)	0 (0.0)	0 (0.0)
Constipation	7 (36.8)	1 (5.3)	0 (0.0)	0 (0.0)	0 (0.0)
Dysphagia	4 (21.1)	0 (0.0)	1 (5.3)	0 (0.0)	0 (0.0)
Diarrhoea	3 (15.8)	1 (5.3)	0 (0.0)	0 (0.0)	0 (0.0)
Stomatitis	0 (0.0)	4 (21.1)	0 (0.0)	0 (0.0)	0 (0.0)
Abdominal discomfort	0 (0.0)	1 (5.3)	0 (0.0)	0 (0.0)	0 (0.0)
Abdominal pain	1 (5.3)	0 (0.0)	0 (0.0)	0 (0.0)	0 (0.0)
Upper abdominal pain	1 (5.3)	0 (0.0)	0 (0.0)	0 (0.0)	0 (0.0)
Indigestion	0 (0.0)	1 (5.3)	0 (0.0)	0 (0.0)	0 (0.0)
Gastroesophageal reflux disease	1 (5.3)	0 (0.0)	0 (0.0)	0 (0.0)	0 (0.0)
Haemorrhoids	0 (0.0)	1 (5.3)	0 (0.0)	0 (0.0)	0 (0.0)
Periodontal disease	1 (5.3)	0 (0.0)	0 (0.0)	0 (0.0)	0 (0.0)
Anal bleeding	0 (0.0)	1 (5.3)	0 (0.0)	0 (0.0)	0 (0.0)
Anal incontinence	0 (0.0)	1 (5.3)	0 (0.0)	0 (0.0)	0 (0.0)
Laboratory test	0 (0.0)	4 (21.1)	9 (47.4)	5 (26.3)	0 (0.0)

Decreased lymphocyte count	0 (0.0)	2 (10.5)	7 (36.8)	5 (26.3)	0 (0.0)
Decreased white blood cell count	1 (5.3)	7 (36.8)	4 (21.1)	0 (0.0)	0 (0.0)
Weight loss	3 (15.8)	4 (21.1)	3 (15.8)	0 (0.0)	0 (0.0)
Decreased platelet count	7 (36.8)	2 (10.5)	0 (0.0)	0 (0.0)	0 (0.0)
Neutrophil count decreased	0 (0.0)	5 (26.3)	3 (15.8)	0 (0.0)	0 (0.0)
Increased white blood cell count	4 (21.1)	0 (0.0)	0 (0.0)	0 (0.0)	0 (0.0)
Elevated liver function test levels	1 (5.3)	2 (10.5)	0 (0.0)	0 (0.0)	0 (0.0)
Alanine aminotransferase increase	2 (10.5)	0 (0.0)	0 (0.0)	0 (0.0)	0 (0.0)
Increased C-reactive protein	1 (5.3)	1 (5.3)	0 (0.0)	0 (0.0)	0 (0.0)
Increased γ-glutamyltransferase	2 (10.5)	0 (0.0)	0 (0.0)	0 (0.0)	0 (0.0)
Increase relative to international standards	2 (10.5)	0 (0.0)	0 (0.0)	0 (0.0)	0 (0.0)
Increased neutrophil count	2 (10.5)	0 (0.0)	0 (0.0)	0 (0.0)	0 (0.0)
Weight gain	1 (5.3)	1 (5.3)	0 (0.0)	0 (0.0)	0 (0.0)
Aspartate aminotransferase increase	1 (5.3)	0 (0.0)	0 (0.0)	0 (0.0)	0 (0.0)
Increased blood bilirubin	1 (5.3)	0 (0.0)	0 (0.0)	0 (0.0)	0 (0.0)
Blood lactate dehydrogenase increased	1 (5.3)	0 (0.0)	0 (0.0)	0 (0.0)	0 (0.0)
Abnormal liver function test	0 (0.0)	1 (5.3)	0 (0.0)	0 (0.0)	0 (0.0)
Decreased reticulocyte count	1 (5.3)	0 (0.0)	0 (0.0)	0 (0.0)	0 (0.0)
Increased blood alkaline phosphatase	1 (5.3)	0 (0.0)	0 (0.0)	0 (0.0)	0 (0.0)

Abnormal renal function test	1 (5.3)	0 (0.0)	0 (0.0)	0 (0.0)	0 (0.0)
Positive clostridial test	0 (0.0)	1 (5.3)	0 (0.0)	0 (0.0)	0 (0.0)
Injury, poisoning and procedural complications	8 (42.1)	7 (36.8)	0 (0.0)	0 (0.0)	0 (0.0)
Wound complication	10 (52.6)	3 (15.8)	0 (0.0)	0 (0.0)	0 (0.0)
Fall	3 (15.8)	4 (21.1)	0 (0.0)	0 (0.0)	0 (0.0)
Contusion	0 (0.0)	3 (15.8)	0 (0.0)	0 (0.0)	0 (0.0)
Postprocedural haemorrhage	2 (10.5)	0 (0.0)	0 (0.0)	0 (0.0)	0 (0.0)
Subdural hematoma	1 (5.3)	0 (0.0)	0 (0.0)	0 (0.0)	0 (0.0)
Ligament injury	0 (0.0)	1 (5.3)	0 (0.0)	0 (0.0)	0 (0.0)
Autonomic dysreflexia	1 (5.3)	0 (0.0)	0 (0.0)	0 (0.0)	0 (0.0)
Infections and infestations	0 (0.0)	11 (57.9)	2 (10.5)	0 (0.0)	0 (0.0)
Upper respiratory tract infection	0 (0.0)	7 (36.8)	0 (0.0)	0 (0.0)	0 (0.0)
Gastroenteritis	0 (0.0)	3 (15.8)	0 (0.0)	0 (0.0)	0 (0.0)
Influenza	0 (0.0)	2 (10.5)	0 (0.0)	0 (0.0)	0 (0.0)
Sinusitis	1 (5.3)	1 (5.3)	0 (0.0)	0 (0.0)	0 (0.0)
Tinea cruris	0 (0.0)	2 (10.5)	0 (0.0)	0 (0.0)	0 (0.0)
Cystitis	0 (0.0)	1 (5.3)	0 (0.0)	0 (0.0)	0 (0.0)
Oral candidiasis	0 (0.0)	1 (5.3)	0 (0.0)	0 (0.0)	0 (0.0)
Otitis externa	0 (0.0)	1 (5.3)	0 (0.0)	0 (0.0)	0 (0.0)

Pathogen resistance	0 (0.0)	1 (5.3)	0 (0.0)	0 (0.0)	0 (0.0)
Tinea pedis	0 (0.0)	1 (5.3)	0 (0.0)	0 (0.0)	0 (0.0)
Urinary tract infection	0 (0.0)	1 (5.3)	0 (0.0)	0 (0.0)	0 (0.0)
Vulvitis	1 (5.3)	0 (0.0)	0 (0.0)	0 (0.0)	0 (0.0)
Wound infection	0 (0.0)	0 (0.0)	1 (5.3)	0 (0.0)	0 (0.0)
Tinea infection	0 (0.0)	1 (5.3)	0 (0.0)	0 (0.0)	0 (0.0)
Postprocedural infection	0 (0.0)	0 (0.0)	1 (5.3)	0 (0.0)	0 (0.0)
Metabolism and nutritional disorders	4 (21.1)	1 (5.3)	4 (21.1)	2 (10.5)	0 (0.0)
Loss of appetite	3 (15.8)	2 (10.5)	3 (15.8)	0 (0.0)	0 (0.0)
Hyponatremia	3 (15.8)	0 (0.0)	2 (10.5)	0 (0.0)	0 (0.0)
Hypernatremia	2 (10.5)	0 (0.0)	0 (0.0)	2 (10.5)	0 (0.0)
Hypoalbuminemia	1 (5.3)	1 (5.3)	0 (0.0)	0 (0.0)	0 (0.0)
Dehydration	0 (0.0)	1 (5.3)	0 (0.0)	0 (0.0)	0 (0.0)
Hyperchloremia	1 (5.3)	0 (0.0)	0 (0.0)	0 (0.0)	0 (0.0)
Hypertriglyceridemia	0 (0.0)	1 (5.3)	0 (0.0)	0 (0.0)	0 (0.0)
Hypokalemia	0 (0.0)	0 (0.0)	1 (5.3)	0 (0.0)	0 (0.0)
Hypoproteinemia	0 (0.0)	1 (5.3)	0 (0.0)	0 (0.0)	0 (0.0)
Vascular disorder	0 (0.0)	8 (42.1)	3 (15.8)	0 (0.0)	0 (0.0)
Hypotension	0 (0.0)	8 (42.1)	0 (0.0)	0 (0.0)	0 (0.0)

Hypertension	0 (0.0)	1 (5.3)	3 (15.8)	0 (0.0)	0 (0.0)
Embolism	0 (0.0)	1 (5.3)	0 (0.0)	0 (0.0)	0 (0.0)
Musculoskeletal and connective tissue disorders	3 (15.8)	5 (26.3)	2 (10.5)	0 (0.0)	0 (0.0)
Back pain	4 (21.1)	1 (5.3)	0 (0.0)	0 (0.0)	0 (0.0)
Joint pain	0 (0.0)	3 (15.8)	0 (0.0)	0 (0.0)	0 (0.0)
Muscle weakness	1 (5.3)	0 (0.0)	2 (10.5)	0 (0.0)	0 (0.0)
Musculoskeletal pain	1 (5.3)	2 (10.5)	0 (0.0)	0 (0.0)	0 (0.0)
Limb pain	2 (10.5)	1 (5.3)	0 (0.0)	0 (0.0)	0 (0.0)
Myalgia	2 (10.5)	0 (0.0)	0 (0.0)	0 (0.0)	0 (0.0)
Joint effusion	0 (0.0)	1 (5.3)	0 (0.0)	0 (0.0)	0 (0.0)
Muscle spasms	0 (0.0)	1 (5.3)	0 (0.0)	0 (0.0)	0 (0.0)
Respiratory, thoracic and mediastinal disorders	1 (5.3)	7 (36.8)	0 (0.0)	1 (5.3)	0 (0.0)
Aspiration pneumonia	0 (0.0)	4 (21.1)	0 (0.0)	0 (0.0)	0 (0.0)
Pneumonitis	0 (0.0)	3 (15.8)	0 (0.0)	0 (0.0)	0 (0.0)
Cough	1 (5.3)	0 (0.0)	0 (0.0)	0 (0.0)	0 (0.0)
Epistaxis	1 (5.3)	0 (0.0)	0 (0.0)	0 (0.0)	0 (0.0)
Respiratory failure	0 (0.0)	0 (0.0)	0 (0.0)	1 (5.3)	0 (0.0)
Airway hemorrhage	0 (0.0)	1 (5.3)	0 (0.0)	0 (0.0)	0 (0.0)
Allergic rhinitis	1 (5.3)	0 (0.0)	0 (0.0)	0 (0.0)	0 (0.0)

Wheezing	1 (5.3)	0 (0.0)	0 (0.0)	0 (0.0)	0 (0.0)
Cardiac disorder	0 (0.0)	7 (36.8)	1 (5.3)	0 (0.0)	0 (0.0)
Sinus bradycardia	0 (0.0)	6 (31.6)	0 (0.0)	0 (0.0)	0 (0.0)
Sinus tachycardia	0 (0.0)	2 (10.5)	1 (5.3)	0 (0.0)	0 (0.0)
Mental disorder	4 (21.1)	4 (21.1)	0 (0.0)	0 (0.0)	0 (0.0)
Insomnia	5 (26.3)	1 (5.3)	0 (0.0)	0 (0.0)	0 (0.0)
Delirium	0 (0.0)	1 (5.3)	0 (0.0)	0 (0.0)	0 (0.0)
Delusion	0 (0.0)	1 (5.3)	0 (0.0)	0 (0.0)	0 (0.0)
Depression	0 (0.0)	1 (5.3)	0 (0.0)	0 (0.0)	0 (0.0)
Neurosis	0 (0.0)	1 (5.3)	0 (0.0)	0 (0.0)	0 (0.0)
Renal and urinary tract disorders	1 (5.3)	7 (36.8)	0 (0.0)	0 (0.0)	0 (0.0)
Urinary incontinence	0 (0.0)	5 (26.3)	0 (0.0)	0 (0.0)	0 (0.0)
Pollakiuria	1 (5.3)	1 (5.3)	0 (0.0)	0 (0.0)	0 (0.0)
Tonic bladder	0 (0.0)	1 (5.3)	0 (0.0)	0 (0.0)	0 (0.0)
Skin and subcutaneous tissue disorders	3 (15.8)	5 (26.3)	0 (0.0)	0 (0.0)	0 (0.0)
Decubitus ulcer	1 (5.3)	2 (10.5)	0 (0.0)	0 (0.0)	0 (0.0)
Cellulitis	0 (0.0)	2 (10.5)	0 (0.0)	0 (0.0)	0 (0.0)
Dermatitis	0 (0.0)	2 (10.5)	0 (0.0)	0 (0.0)	0 (0.0)
Maculopapular rash	1 (5.3)	1 (5.3)	0 (0.0)	0 (0.0)	0 (0.0)

Contact dermatitis	1 (5.3)	0 (0.0)	0 (0.0)	0 (0.0)	0 (0.0)
Dry skin	0 (0.0)	1 (5.3)	0 (0.0)	0 (0.0)	0 (0.0)
Pruritus	1 (5.3)	0 (0.0)	0 (0.0)	0 (0.0)	0 (0.0)
Redman syndrome	1 (5.3)	0 (0.0)	0 (0.0)	0 (0.0)	0 (0.0)
Seborrheic dermatitis	0 (0.0)	1 (5.3)	0 (0.0)	0 (0.0)	0 (0.0)
Subcutaneous abscess	0 (0.0)	1 (5.3)	0 (0.0)	0 (0.0)	0 (0.0)
Eye disorder	2 (10.5)	3 (15.8)	1 (5.3)	0 (0.0)	0 (0.0)
Asthenopia	0 (0.0)	1 (5.3)	0 (0.0)	0 (0.0)	0 (0.0)
Cataract	0 (0.0)	0 (0.0)	1 (5.3)	0 (0.0)	0 (0.0)
Conjunctival hemorrhage	1 (5.3)	0 (0.0)	0 (0.0)	0 (0.0)	0 (0.0)
Eye discharge	0 (0.0)	1 (5.3)	0 (0.0)	0 (0.0)	0 (0.0)
Photopsia	1 (5.3)	0 (0.0)	0 (0.0)	0 (0.0)	0 (0.0)
Loss of vision	0 (0.0)	1 (5.3)	0 (0.0)	0 (0.0)	0 (0.0)
Blood and lymphatic system disorders	3 (15.8)	2 (10.5)	0 (0.0)	0 (0.0)	0 (0.0)
Anemia	3 (15.8)	2 (10.5)	0 (0.0)	0 (0.0)	0 (0.0)
Ear and labyrinth disorders	3 (15.8)	1 (5.3)	0 (0.0)	0 (0.0)	0 (0.0)
Positional vertigo	1 (5.3)	1 (5.3)	0 (0.0)	0 (0.0)	0 (0.0)
Sensorineural deafness	1 (5.3)	0 (0.0)	0 (0.0)	0 (0.0)	0 (0.0)
Vertigo	1 (5.3)	0 (0.0)	0 (0.0)	0 (0.0)	0 (0.0)

Immune system disorder	1 (5.3)	1 (5.3)	0 (0.0)	0 (0.0)	0 (0.0)
Hypersensitivity	1 (5.3)	1 (5.3)	0 (0.0)	0 (0.0)	0 (0.0)
Neoplasms benign, malignant and unspecified (including cysts and polyps)	2 (10.5)	0 (0.0)	0 (0.0)	0 (0.0)	0 (0.0)
Intracranial tumor hemorrhage	2 (10.5)	0 (0.0)	0 (0.0)	0 (0.0)	0 (0.0)
Endocrine disorder	0 (0.0)	1 (5.3)	0 (0.0)	0 (0.0)	0 (0.0)
Diabetes insipidus	0 (0.0)	1 (5.3)	0 (0.0)	0 (0.0)	0 (0.0)

Physician terms are coded using CTCAE v 4.03. Multiple events in the same SOC or PT were counted only once at the highest grade.

Figure S1. Histology of biopsy specimens before each G47Δ administration for each patient (n=19)

Biopsies were performed immediately before each G47Δ injection, and G47Δ was injected to coordinates different from prior injections. In general, $CD4^+$ and $CD8^+$ lymphocytes within the tumor increased in number as G47Δ injections were repeated, whereas the number of $Foxp3^+$ cells remained low throughout. Each is representative of 4 biopsy specimens.

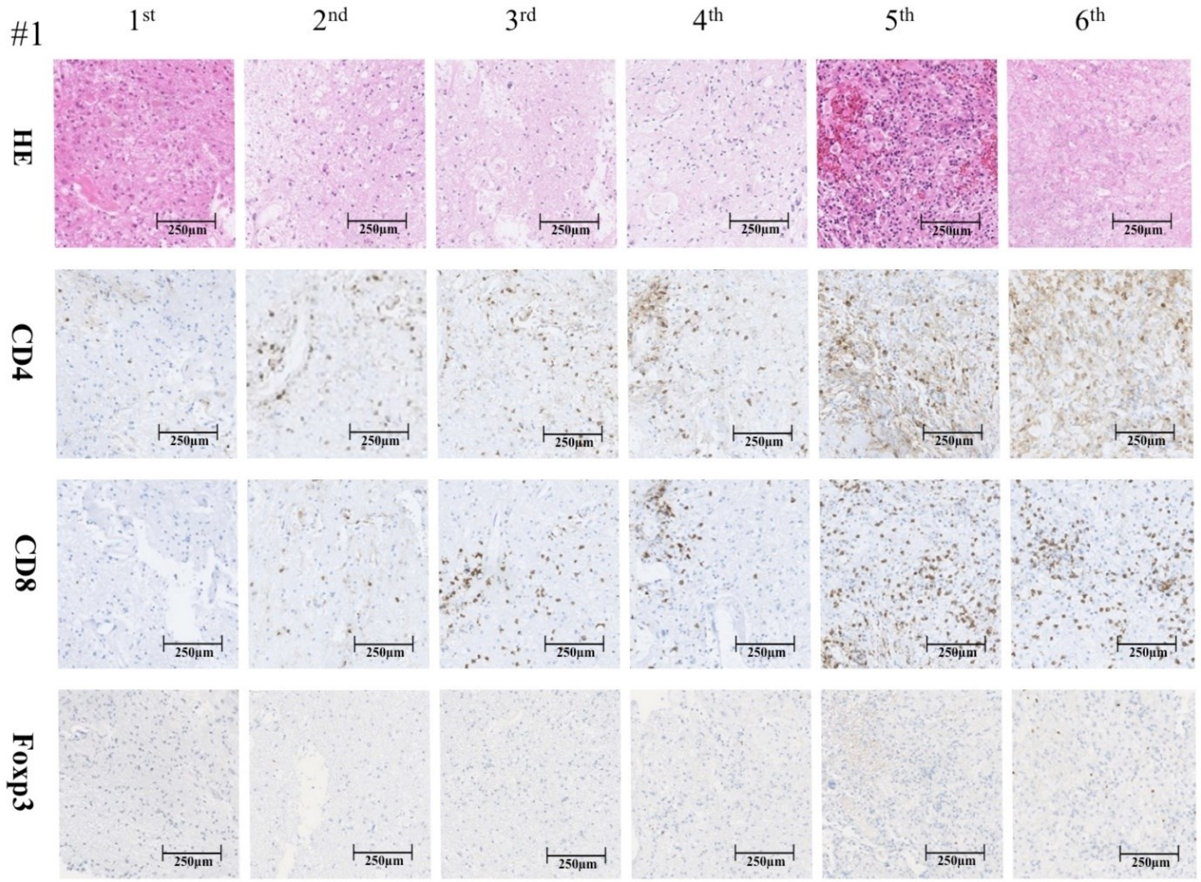

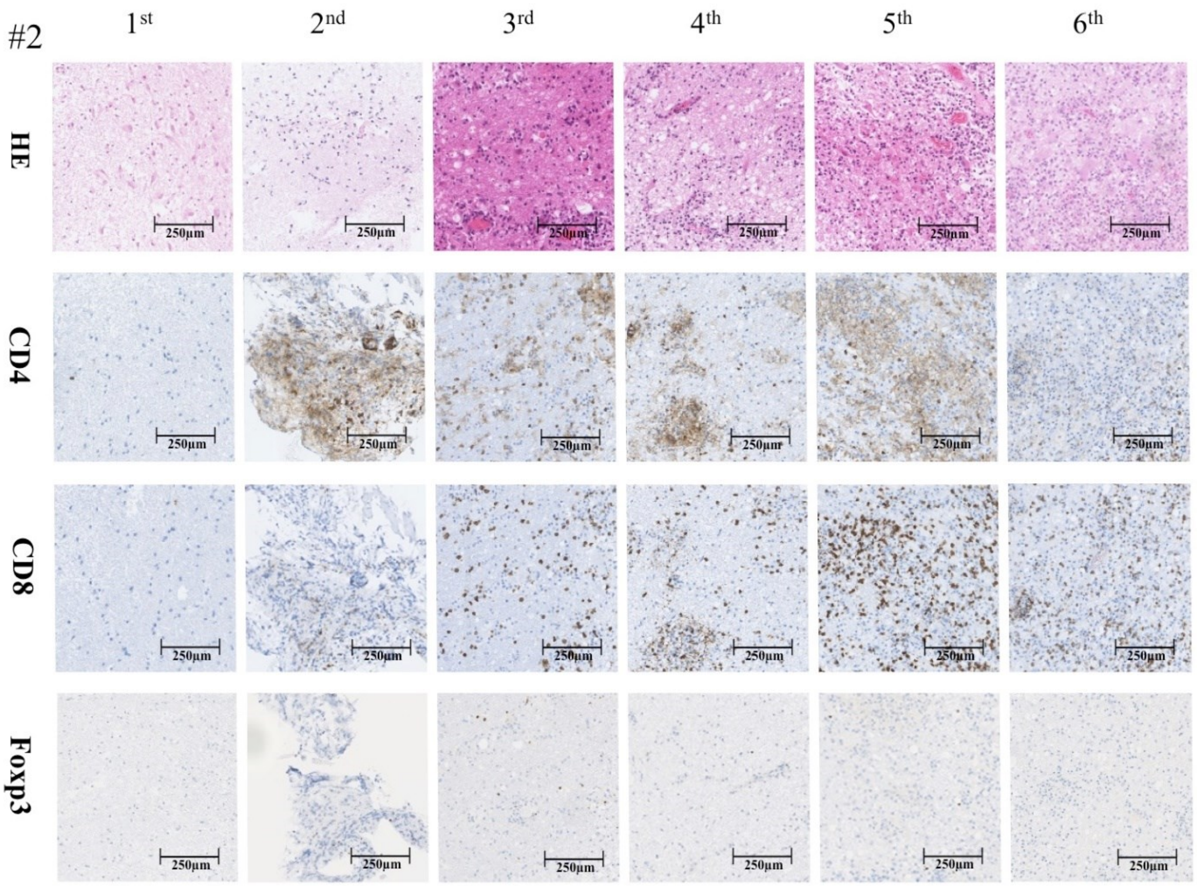
#2
1st
2nd
3rd
4th
5th
6th
HE
CD4
CD8
Foxp3
250μm

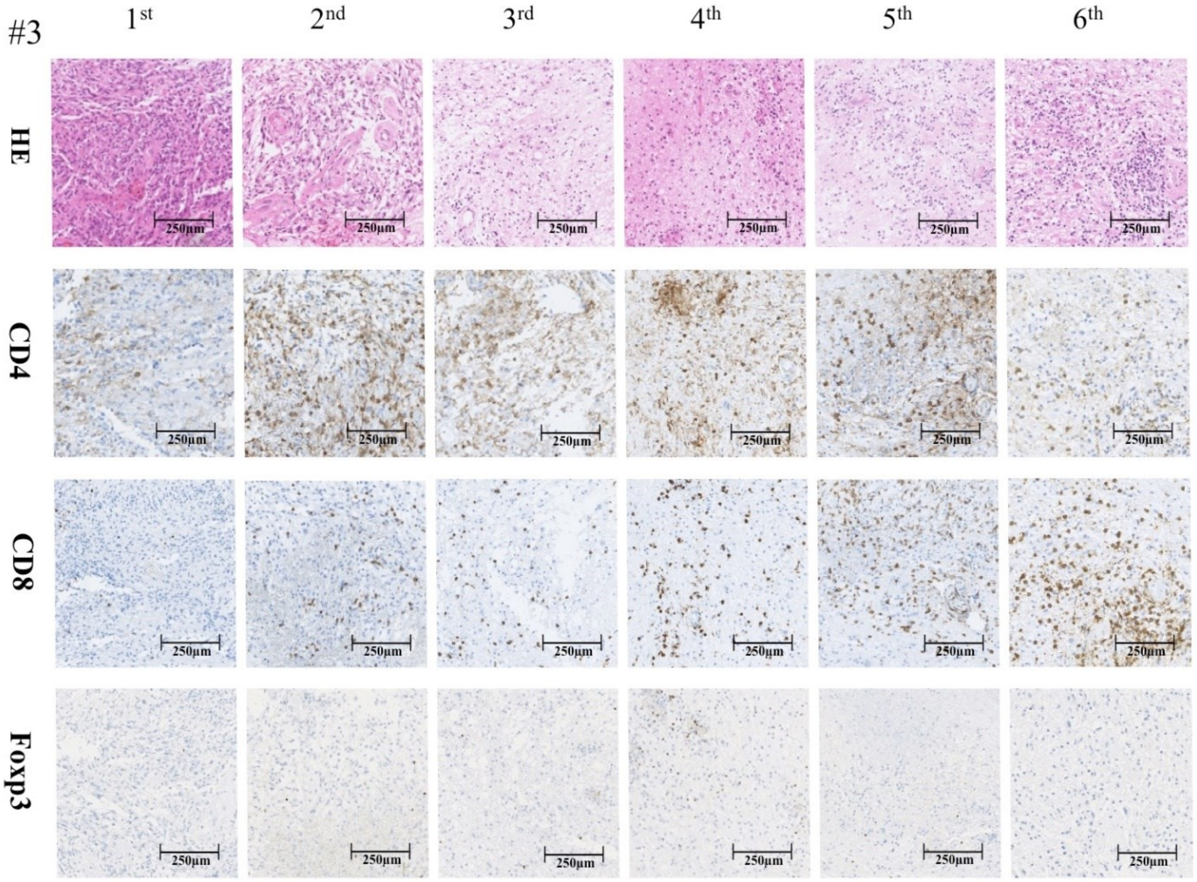
#3
1st
2nd
3rd
4th
5th
6th
HE
CD4
CD8
Foxp3
250μm

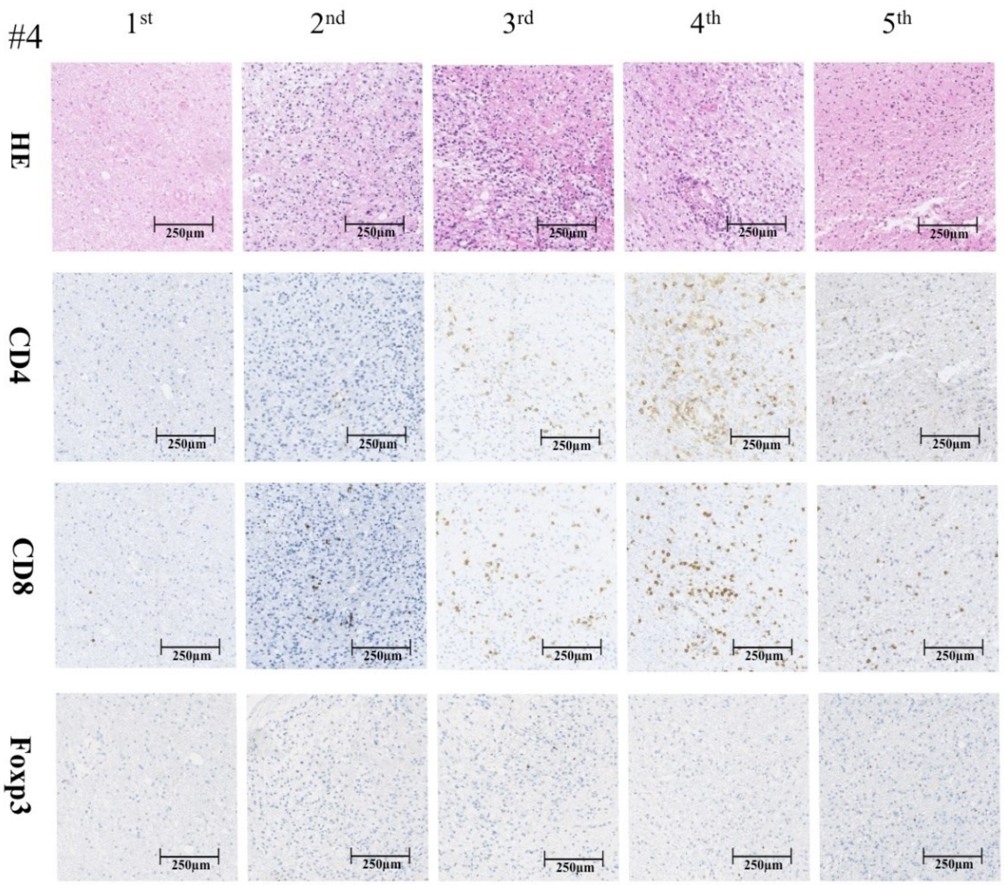
#4
1st
2nd
3rd
4th
5th
HE
CD4
CD8
Foxp3
250μm

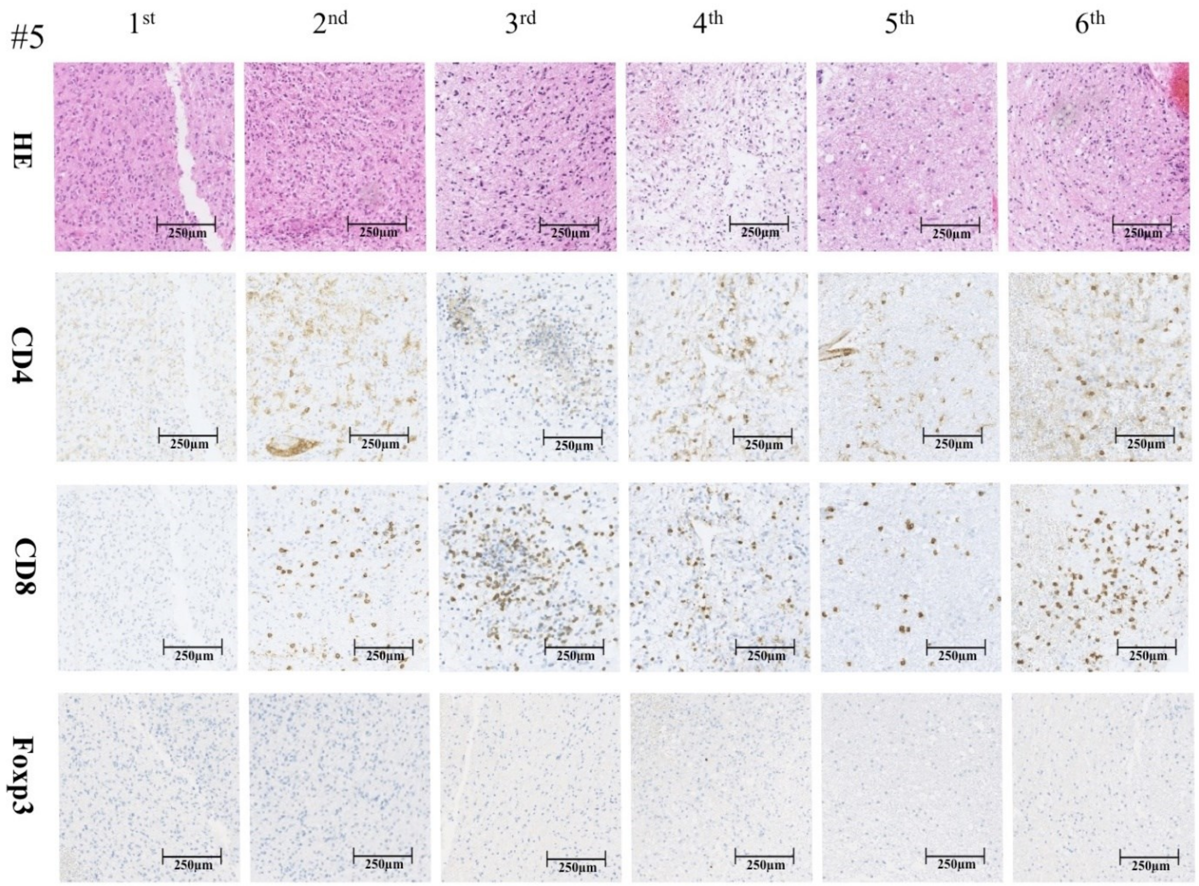
#5
1st
2nd
3rd
4th
5th
6th
HE
CD4
CD8
Foxp3
250μm

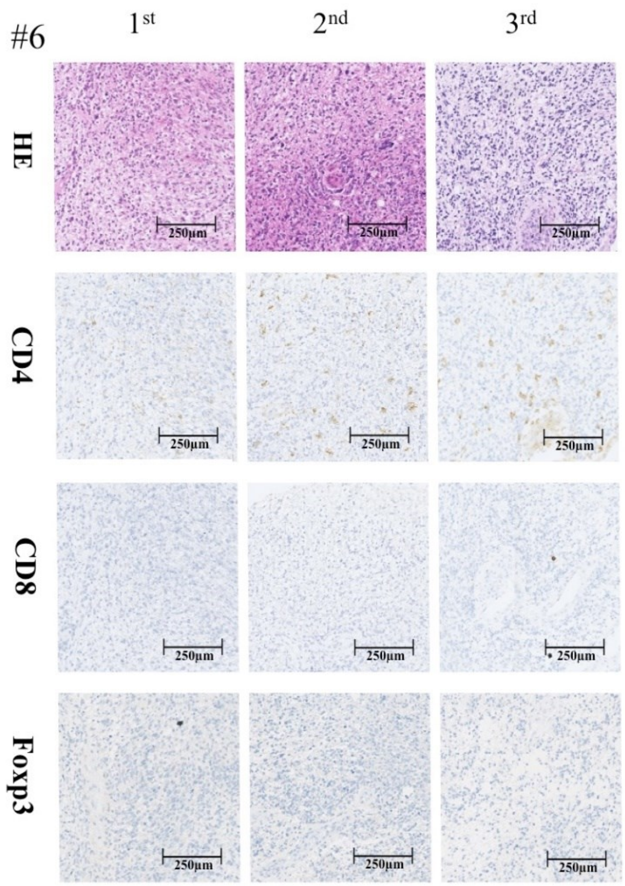
#6
1st
2nd
3rd
HE
CD4
CD8
Foxp3
250µm

#7

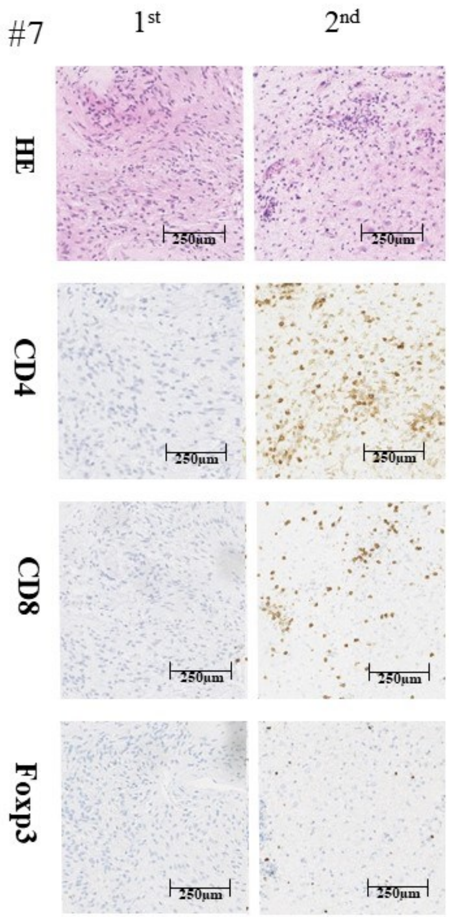

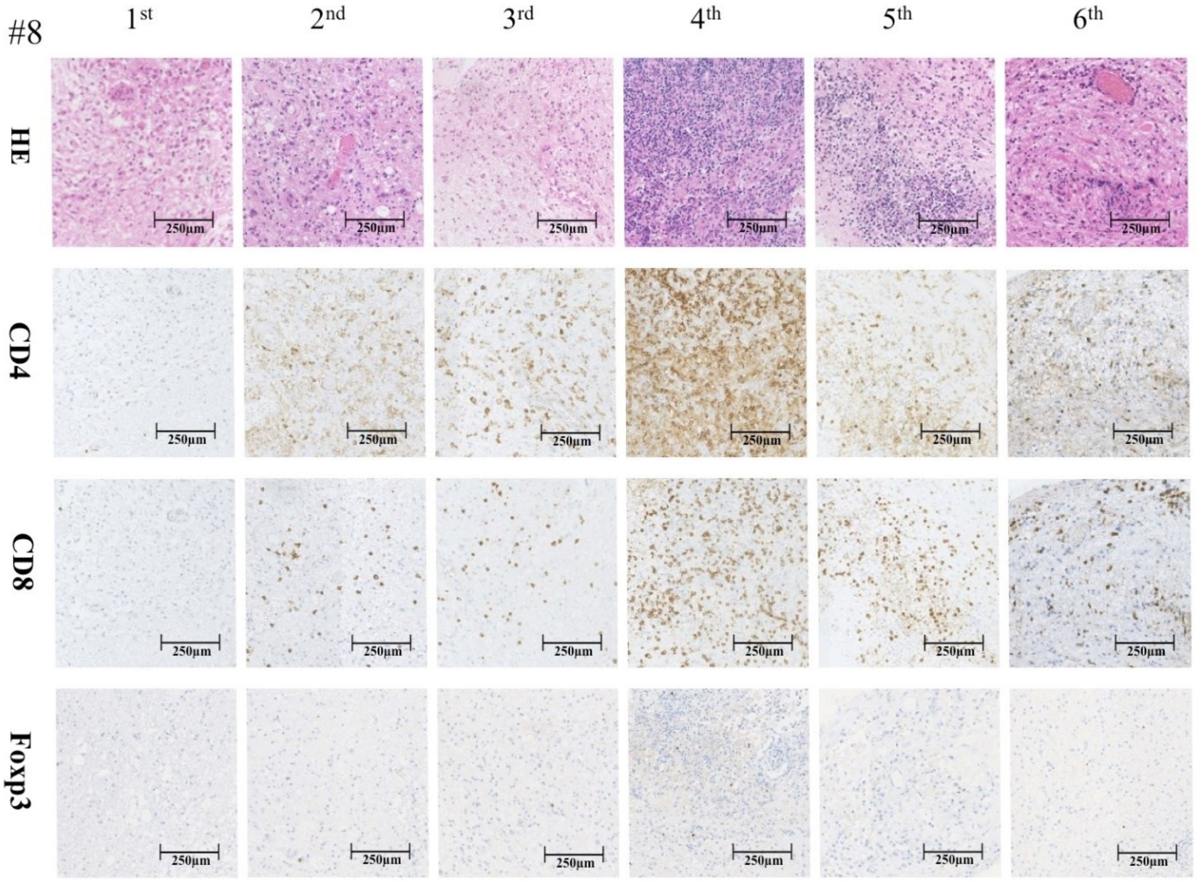
#8
1st
2nd
3rd
4th
5th
6th
HE
CD4
CD8
Foxp3
250µm

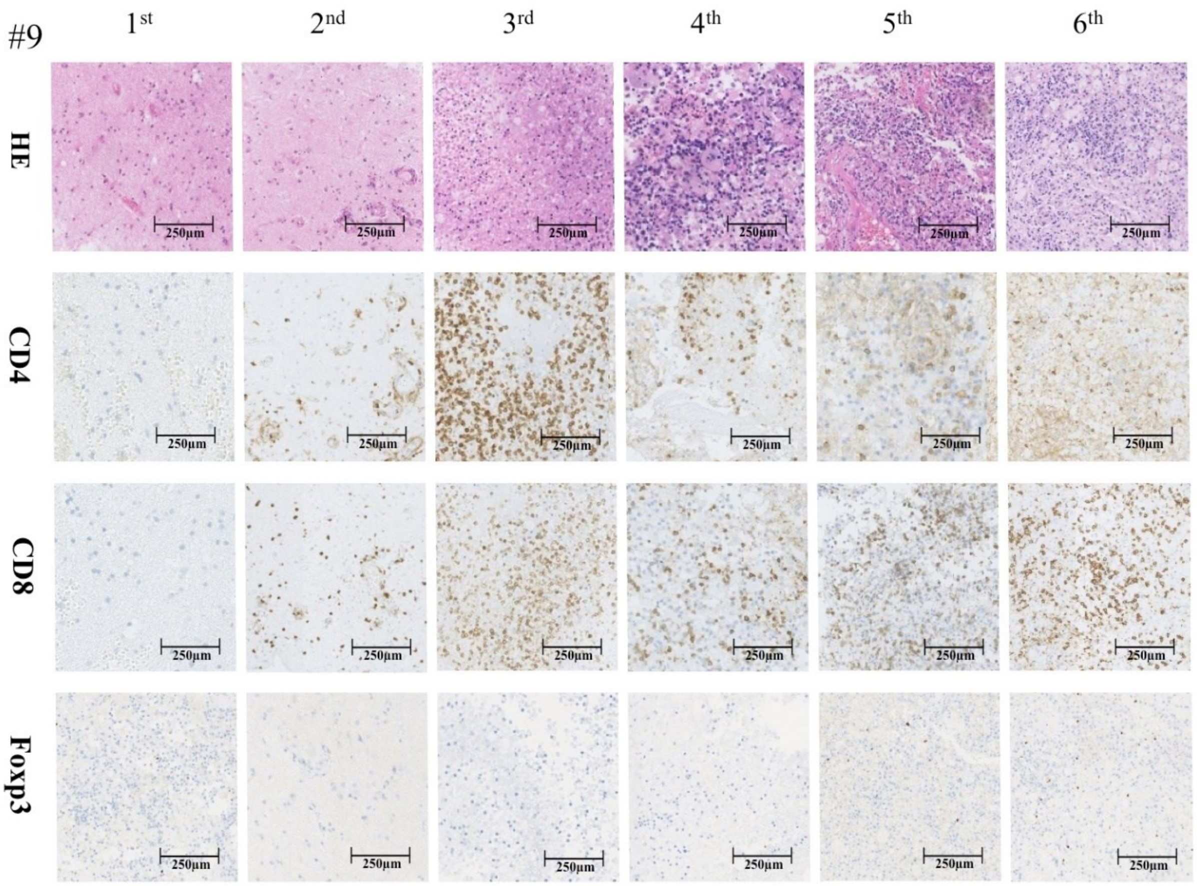
#9
1st
2nd
3rd
4th
5th
6th
HE
CD4
CD8
Foxp3
250µm

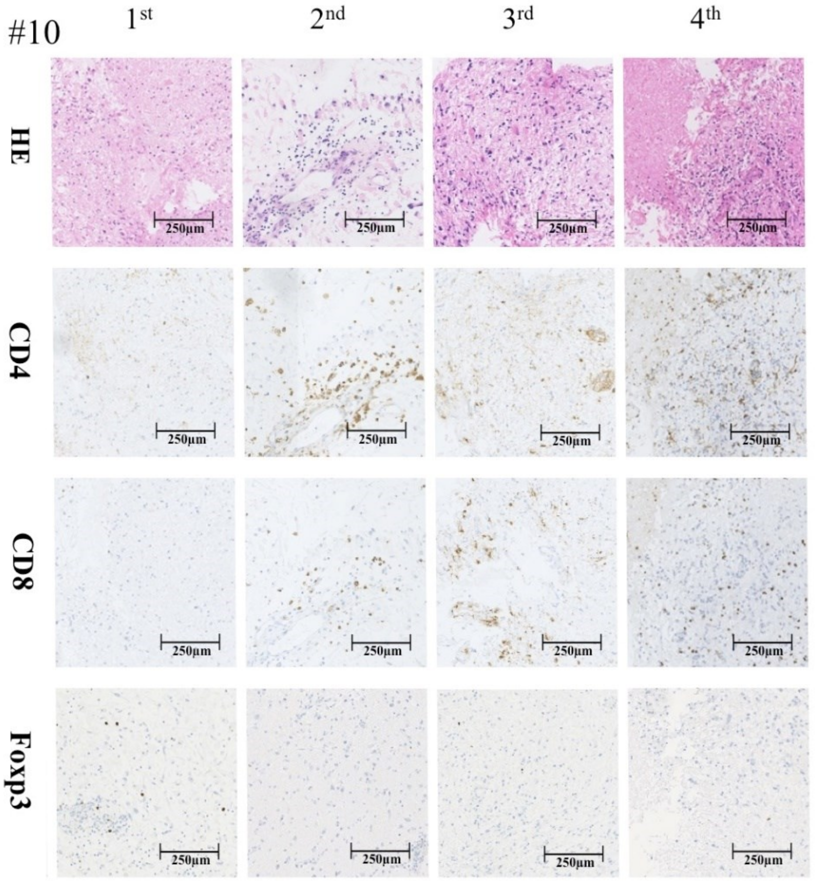
#10
1st
2nd
3rd
4th
HE
CD4
CD8
Foxp3
250μm

#11

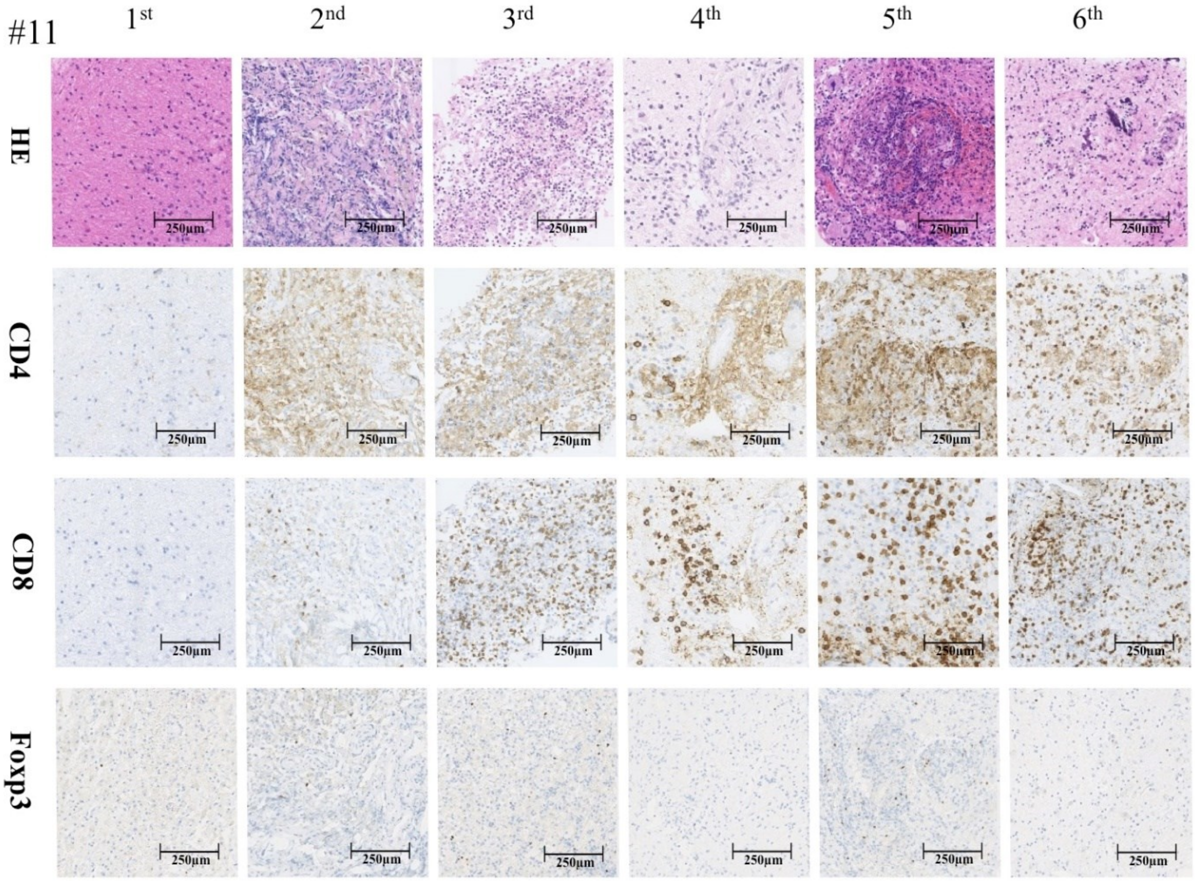

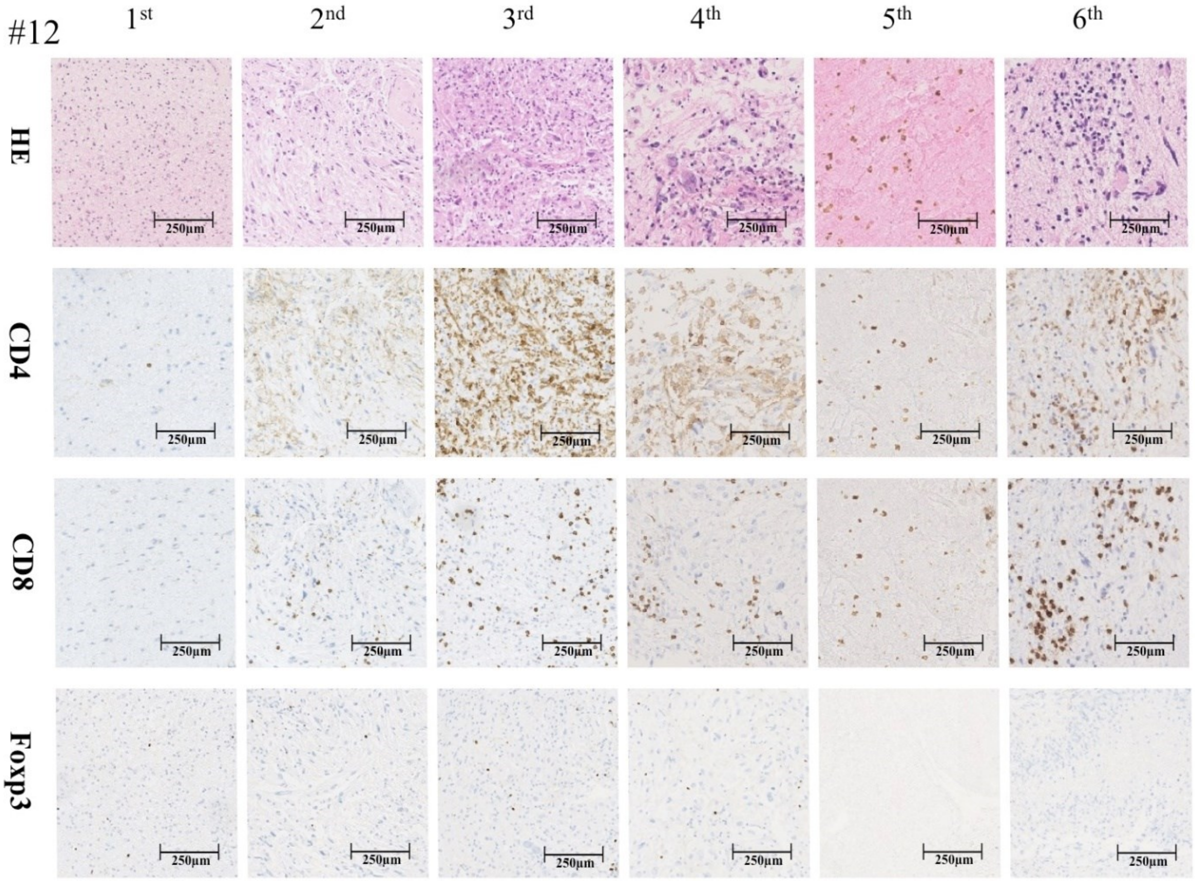
#12
1st
2nd
3rd
4th
5th
6th
HE
CD4
CD8
Foxp3
250μm

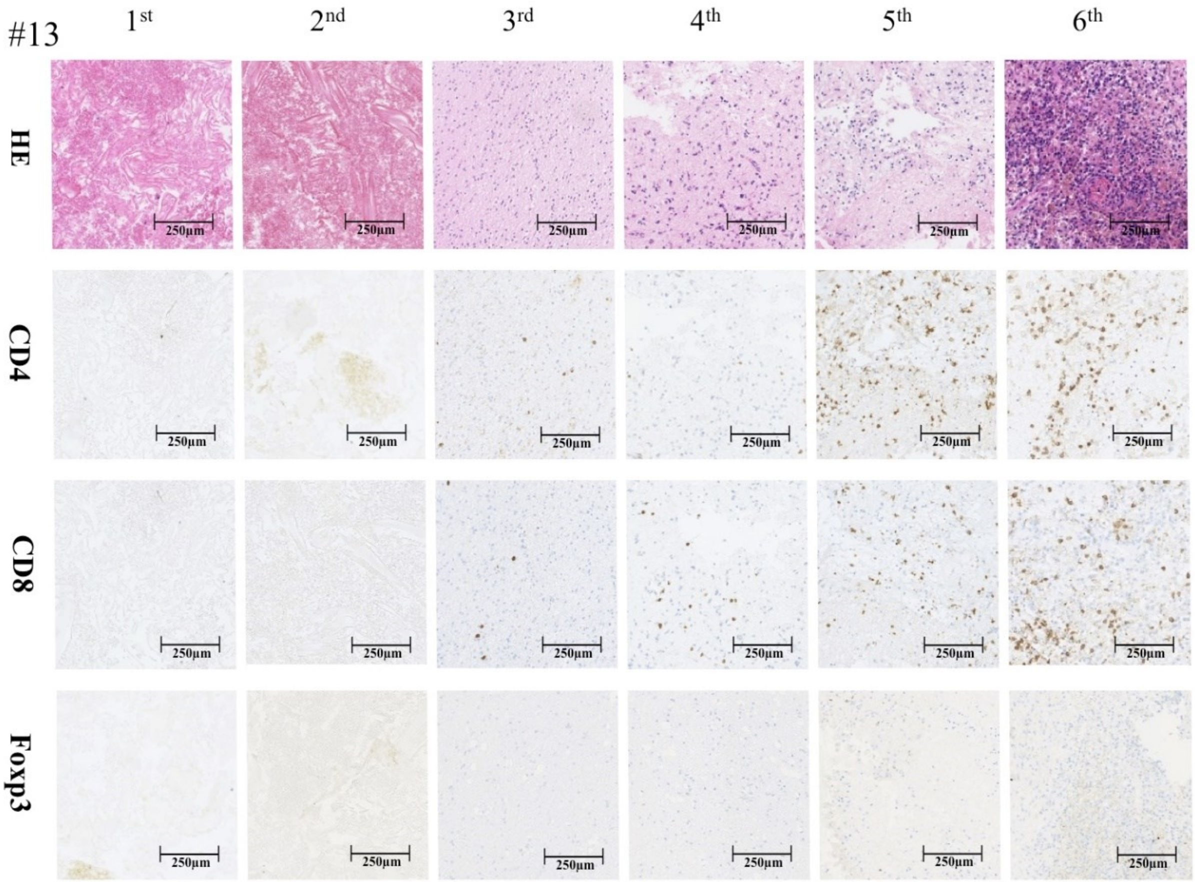

#13
1st
2nd
3rd
4th
5th
6th
HE
CD4
CD8
Foxp3
250μm

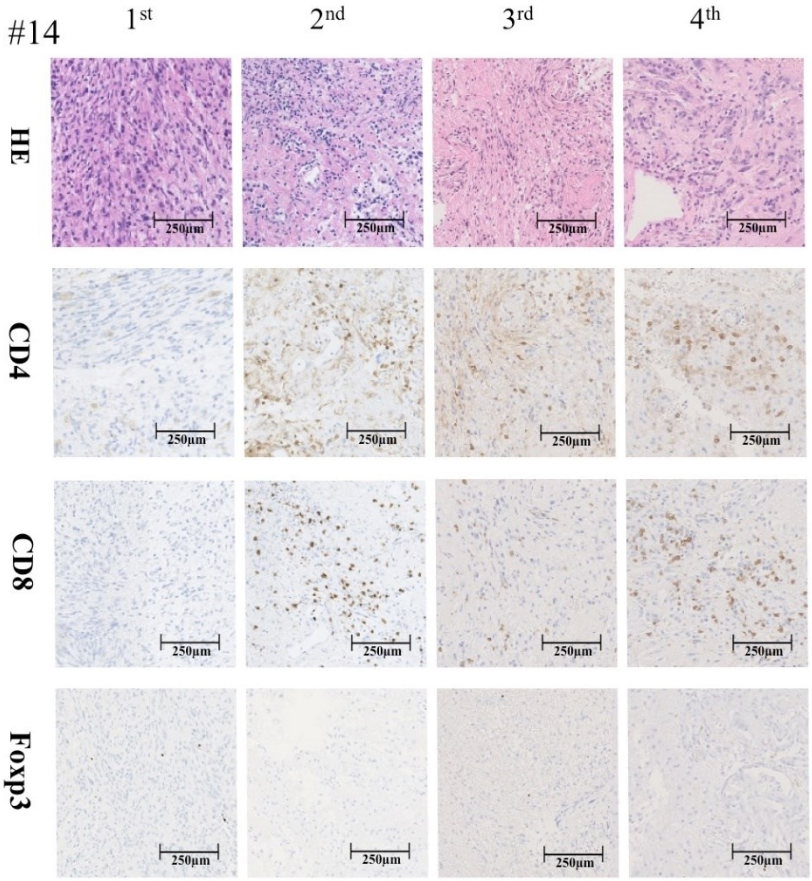

#14
1st
2nd
3rd
4th
HE
CD4
CD8
Foxp3
250μm

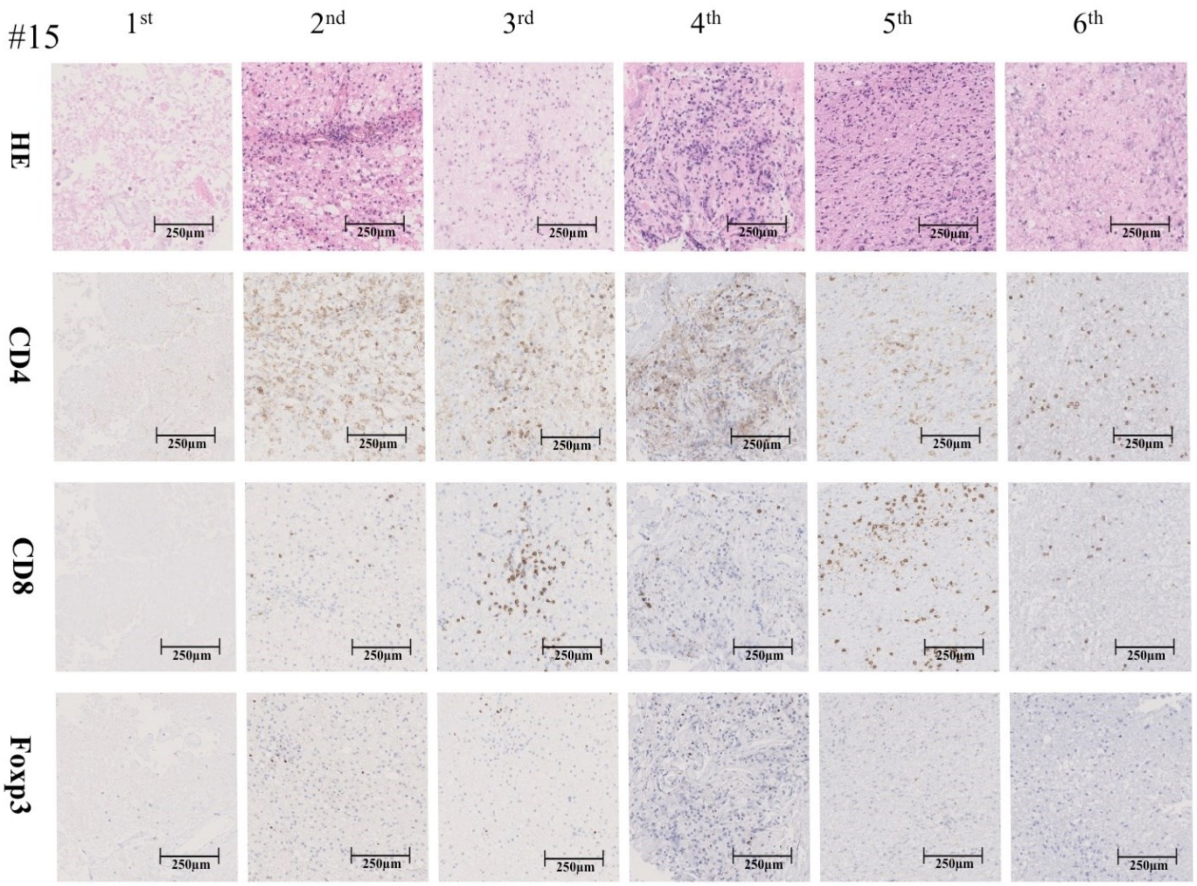
#15
1st
2nd
3rd
4th
5th
6th
HE
CD4
CD8
Foxp3
250μm

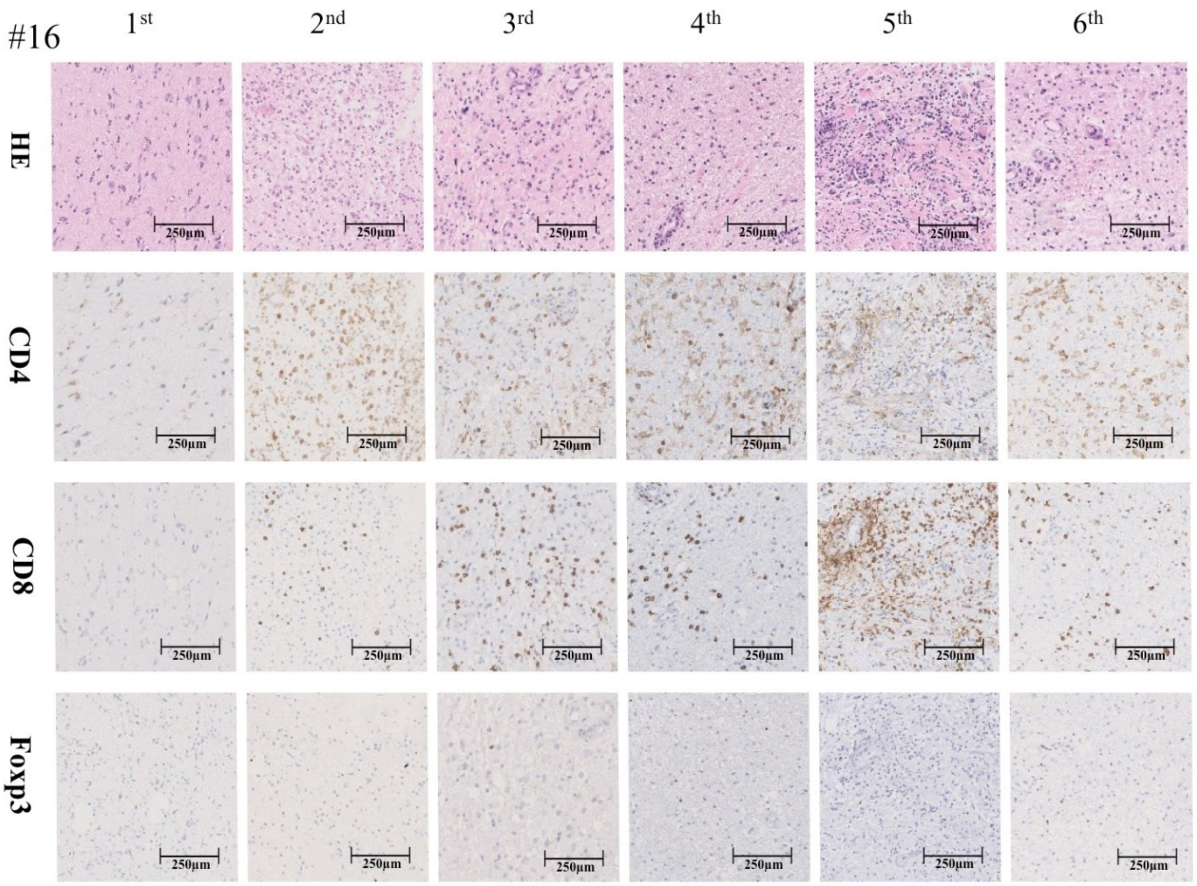
#16
1st
2nd
3rd
4th
5th
6th
HE
CD4
CD8
Foxp3
250μm

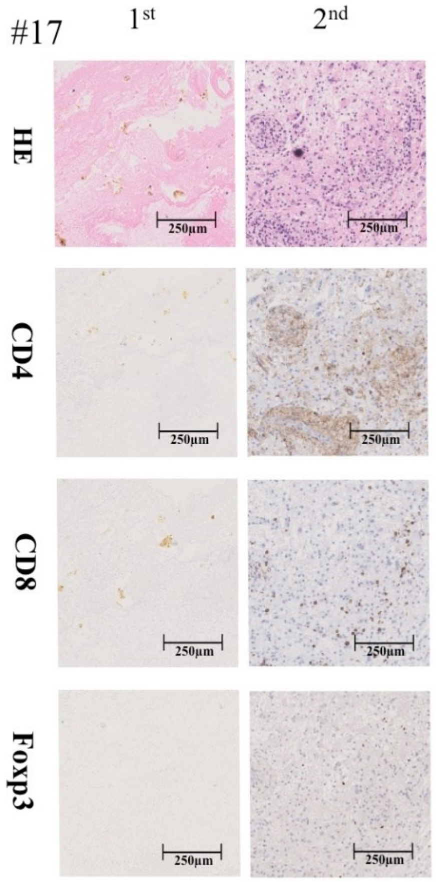
#17
1st
2nd
HE
CD4
CD8
Foxp3
250μm

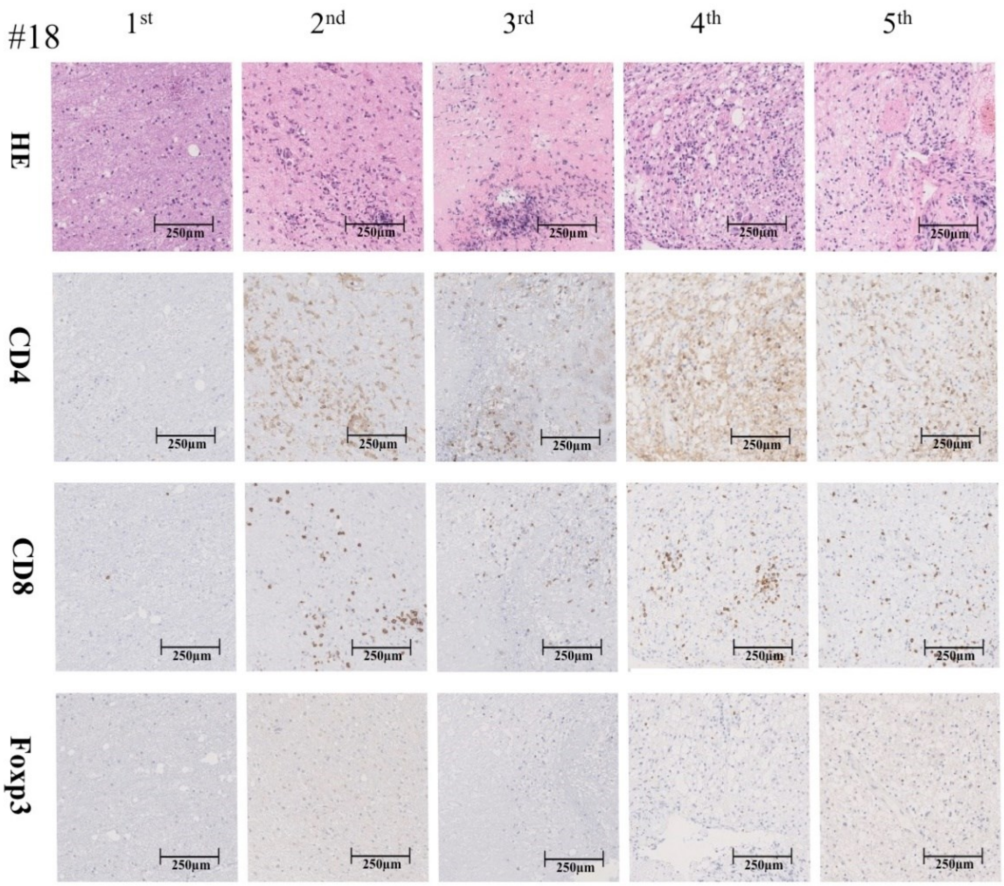
#18
1st
2nd
3rd
4th
5th
HE
CD4
CD8
Foxp3
250μm

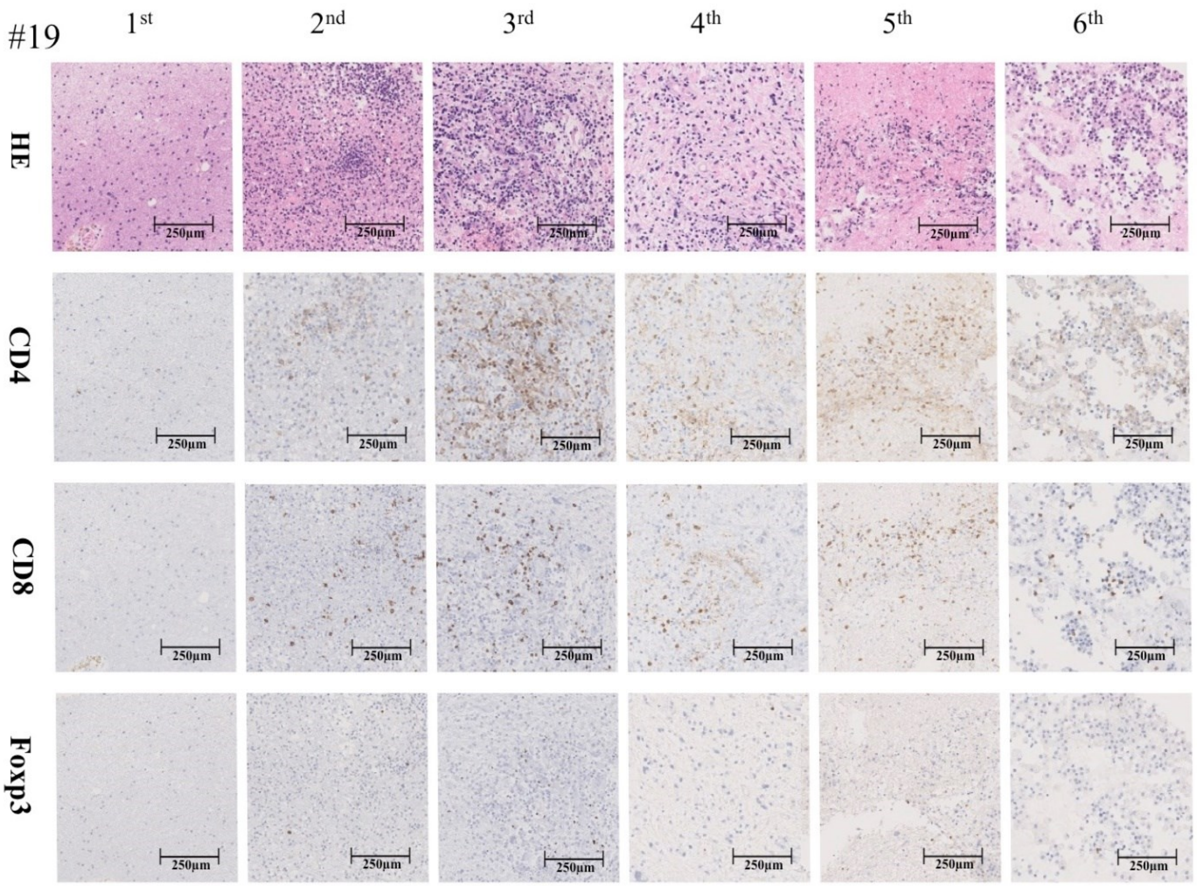
#19
1st
2nd
3rd
4th
5th
6th
HE
CD4
CD8
Foxp3
250μm
