# Supplementary material for: Intratumoral oncolytic herpes virus G47∆ for residual or recurrent glioblastoma: a phase 2 trial
Source: Nat Med. 2022 Jul 21;28(8):1630–9. doi: 10.1038/s41591-022-01897-x (PMC9388376; doi:10.1038/s41591-022-01897-x)
Supplement: Supplementary file 2 — Reporting Summary [file 41591_2022_1897_MOESM2_ESM.pdf]

nature research

Corresponding author(s): Tomoki Todo

Last updated by author(s): 2022/5/26

Reporting Summary

Nature Research wishes to improve the reproducibility of the work that we publish. This form provides structure for consistency and transparency in reporting. For further information on Nature Research policies, see our Editorial Policies and the Editorial Policy Checklist.

Statistics

For all statistical analyses, confirm that the following items are present in the figure legend, table legend, main text, or Methods section.

n/a	Confirmed	
☐	☒	The exact sample size (*n*) for each experimental group/condition, given as a discrete number and unit of measurement
☐	☒	A statement on whether measurements were taken from distinct samples or whether the same sample was measured repeatedly
☐	☒	The statistical test(s) used AND whether they are one- or two-sided<br>*Only common tests should be described solely by name; describe more complex techniques in the Methods section.*
☐	☒	A description of all covariates tested
☒	☐	A description of any assumptions or corrections, such as tests of normality and adjustment for multiple comparisons
☐	☒	A full description of the statistical parameters including central tendency (e.g. means) or other basic estimates (e.g. regression coefficient) AND variation (e.g. standard deviation) or associated estimates of uncertainty (e.g. confidence intervals)
☐	☒	For null hypothesis testing, the test statistic (e.g. *F*, *t*, *r*) with confidence intervals, effect sizes, degrees of freedom and *P* value noted<br>*Give P values as exact values whenever suitable.*
☒	☐	For Bayesian analysis, information on the choice of priors and Markov chain Monte Carlo settings
☒	☐	For hierarchical and complex designs, identification of the appropriate level for tests and full reporting of outcomes
☒	☐	Estimates of effect sizes (e.g. Cohen's *d*, Pearson's *r*), indicating how they were calculated

Our web collection on statistics for biologists contains articles on many of the points above.

Software and code

Policy information about availability of computer code

Data collection	SAS Windows, version 9.4 (SAS Institute Inc., Cary, NC, USA)
Data analysis	IBM SPSS Statistics version 22 software (IBM Corporation, Somers, USA)

For manuscripts utilizing custom algorithms or software that are central to the research but not yet described in published literature, software must be made available to editors and reviewers. We strongly encourage code deposition in a community repository (e.g. GitHub). See the Nature Research guidelines for submitting code & software for further information.

Data

Policy information about availability of data

All manuscripts must include a data availability statement. This statement should provide the following information, where applicable:

- Accession codes, unique identifiers, or web links for publicly available datasets
- A list of figures that have associated raw data
- A description of any restrictions on data availability

Any requests for raw and analyzed data will be reviewed by the Institute of Medical Science Hospital, the University of Tokyo. Patient-related data not included in the paper were generated as part of a clinical trial and are subject to patient confidentiality. Any data and materials (for example, tissue samples or imaging data) that can be shared will need approval from the Institute of Medical Science Hospital, the University of Tokyo. Any data shared will be de-identified. Requests should be made to Tomoki Todo (toudou-nsu@umin.ac.jp); response time will be within approximately 5-10 business days.

Field-specific reporting

Please select the one below that is the best fit for your research. If you are not sure, read the appropriate sections before making your selection.

☒ Life sciences ☐ Behavioural & social sciences ☐ Ecological, evolutionary & environmental sciences

For a reference copy of the document with all sections, see nature.com/documents/nr-reporting-summary-flat.pdf

Life sciences study design

All studies must disclose on these points even when the disclosure is negative.

Sample size	At planning, this trial assumed achievement of a 1-year survival rate of 40% based on the results of the FIH trial2. Based on the 1-year survival rate for recurrent glioblastoma after chemo-radiotherapy (14%)3, the comparative control value was set to 15%. Assuming a superiority of G47Δ of 5% on one side and a power of 80%, and one interim analysis to be performed, the treatment arm size was calculated as 25 patients. An interim analysis was to be conducted when the number of patients followed for 1 year from the initiation of study treatment reached 13 patients, so the study was designed to include 30 patients to ensure adequate enrollment. The increase in the number of Type I errors resulting from the interim analysis will be adjusted by the Lan-Demet's method using the O'Brien Fleming type α. The significance level of the hypothesis test will be set at 0.557% on one side. Efficacy and safety analyses included all patients who received at least one dose of G47Δ and this defined the full analysis set (FAS) for efficacy and the safety analysis set (SAS) for safety.
Data exclusions	Patients were excluded based on a priori criteria before the start of the study. There were no data excluded for enrolled patients.
Replication	Findings were from a cohort of patients with glioblastoma, all but 3 of whom are now deceased; replication in this cohort is therefore impossible.
Randomization	This was a phase II open-label study without randomization. Randomization was not feasible due to the nature of the surgical intervention and ethical restrictions related to 'sham' surgery in Japan.
Blinding	Blinding not conducted. Open-label study design using surgical patients made blinding not relevant.

Reporting for specific materials, systems and methods

We require information from authors about some types of materials, experimental systems and methods used in many studies. Here, indicate whether each material, system or method listed is relevant to your study. If you are not sure if a list item applies to your research, read the appropriate section before selecting a response.

Materials & experimental systems

n/a	Involved in the study	
☐	☒	Antibodies
☒	☐	Eukaryotic cell lines
☒	☐	Palaeontology and archaeology
☒	☐	Animals and other organisms
☐	☒	Human research participants
☐	☒	Clinical data
☒	☐	Dual use research of concern

Methods

n/a	Involved in the study	
☒	☐	ChIP-seq
☒	☐	Flow cytometry
☐	☒	MRI-based neuroimaging

Antibodies

Antibodies used	Antibodies used<br>Anti-CD4 (rabbit) (clone EPR6855, Abcam, Cat. ab133616, Lot. GR3276764-5, dilution 1:250)<br>Anti-CD8 (rabbit) (clone SP16, Abcam, Cat. ab101500, Lot. 9116S1711F, dilution 1:100)<br>Anti-FoxP3 (rabbit) (clone SP97, Abcam, Cat. ab99963, Lot. GR3281127-15, dilution 1:50)<br>Anti-HSV-1 (rabbit) (polyclonal, Gene Tex, Cat. GTX73373, Lot. 822100458, ready-to-use)<br>Anti-IDH1 R132H (mouse) (clone H09, Dianova, Cat. DIA-H09, Lot. 211129/02, dilution 1:100)<br>Anti-MGMT (mouse) (clone MT3.1, Abcam, Cat. ab39253, Lot. GR3422909-2, dilution 1:100)
Validation	All antibodies were purchased directly from manufacturers ana the validation statements are available on website of the manufacturers. The application of all antibodies followed by the instructions of the website.<br>Anti-CD4 (ab133616): https://www.abcam.com/cd4-antibody-epr6855-ab133616.html<br>Anti-CD8 (ab101500): https://www.abcam.com/cd8-alpha-antibody-sp16-ab101500.html<br>Anti-FoxP3 (ab99963): https://www.abcam.com/foxp3-antibody-sp97-ab99963.html<br>Anti-HSV-1 (GTX73373): https://www.genetex.com/Product/Detail/HSV1-antibody-ready-to-use/GTX73373<br>Anti-IDH1 R132H (DIA-H09): https://www.dianova.com/en/shop/dia-h09-anti-idh1-r132h-hu-from-mouse-h09-unconj/

Anti-MGMT (ab39253): https://www.abcam.com/mgmt-antibody-mt31-ab39253.html

Human research participants

Policy information about studies involving human research participants

Population characteristics	A total of 19 patients were included in the study who were diagnosed with residual or recurrent glioblastoma. Covariate-related characteristics of the 19 patients are included in Table 1.
Recruitment	Study participants were enrolled through patient referrals or the website of the Institute of Medical Science Hospital, the University of Tokyo. All eligible patients were enrolled sequentially without exception and intentional selection.
Ethics oversight	All patients enrolled in the trial provided written informed consent. The protocol was approved by the institutional review board of the Institution of Medical Science, the University of Tokyo. Patients were not compensated for trial participation.

Note that full information on the approval of the study protocol must also be provided in the manuscript.

Clinical data

Policy information about clinical studies

All manuscripts should comply with the ICMJE guidelines for publication of clinical research and a completed CONSORT checklist must be included with all submissions.

Clinical trial registration	UMIN-CTR Clinical Trial registry (UMIN000015995)
Study protocol	Full trial protocol is provided in the Supplementary Materials accompanying the main manuscript.
Data collection	Data was collected from patients who underwent study interventions at a single institution (the Institute of Medical Science Hospital, the University of Tokyo [IMSUT Hospital]) in Japan. The first patient enrollment date was May 19, 2015 and the last patient enrollment date was April 18, 2018. This study was approved by the Japanese Pharmaceuticals and Medical Devices Agency (PMDA) on August 29, 2014. Under the guidance of PMDA, it was recommended that this study be conducted at a single institution for safety reasons, as this was the first PMDA-supervised clinical trial of oncolytic virus therapy. The data cutoff date for the Case Study Report submitted to PMDA was April 6, 2020, although survival-related data continued to be collected until March 1, 2022.
Outcomes	The primary endpoint was the 1-year survival rate after G47Δ treatment initiation. For the primary endpoint, a 1-year survival rate after G47Δ initiation was calculated along with the 95% confidence interval.<br>Secondary endpoints included OS, progression-free survival (PFS), tumor response for efficacy and adverse event frequency.<br>Further details are recorded in the main manuscript. For the secondary endpoints, an overall survival after G47Δ initiation, a progression-free survival after G47Δ initiation and an overall survival from the initial surgery with respective 95% confidence intervals were calculated by the Kaplan-Meier method. Adverse events for the safety assessment were analyzed by event.

Magnetic resonance imaging

Experimental design

Design type	MRI with contrast enhancement according to protocol schedules.
Design specifications	MRI with contrast enhancement according to protocol schedules.
Behavioral performance measures	This was not an fMRI study.

Acquisition

Imaging type(s)	Enhanced 3D-T1WI
Field strength	3 Tesla
Sequence & imaging parameters	Standard parameters of the radiology departments of the institutions.
Area of acquisition	Whole brain

Diffusion MRI ☐ Used ☒ Not used

Preprocessing

Preprocessing software	No preprocessing was performed; MR images were directly analyzed in our PACS.
Normalization	N/A based on no preprocessing performed
Normalization template	N/A based on no preprocessing performed

Noise and artifact removal: N/A based on no preprocessing performed

Volume censoring: N/A based on no preprocessing performed

Statistical modeling & inference

Model type and settings: No statistical modeling and inference performed

Effect(s) tested: N/A

Specify type of analysis: [x] Whole brain [ ] ROI-based [ ] Both

Statistic type for inference (See Eklund et al. 2016): N/A

Correction: N/A

Models & analysis

n/a	Involved in the study
[x]	[ ] Functional and/or effective connectivity
[x]	[ ] Graph analysis
[x]	[ ] Multivariate modeling or predictive analysis
