# Supplementary material for: Author Correction: Intratumoral oncolytic herpes virus G47∆ for residual or recurrent glioblastoma: a phase 2 trial
Source: Nat Med. 2025 Mar 18;31(4):1365. doi: 10.1038/s41591-025-03619-5 (PMC12003165; doi:10.1038/s41591-025-03619-5)

nature medicine

Supplementary information

Author Correction: Intratumoral oncolytic herpes virus G47∆ for residual or recurrent glioblastoma: a phase 2 trial

In the format provided by the authors and unedited

Original Fig. 2c

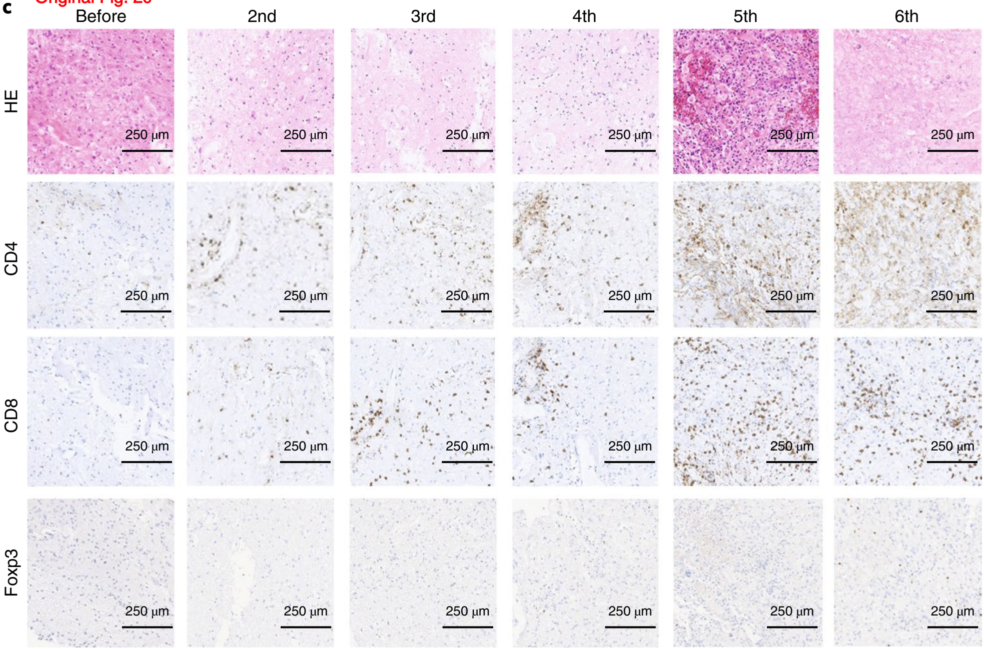

Corrected Fig. 2c

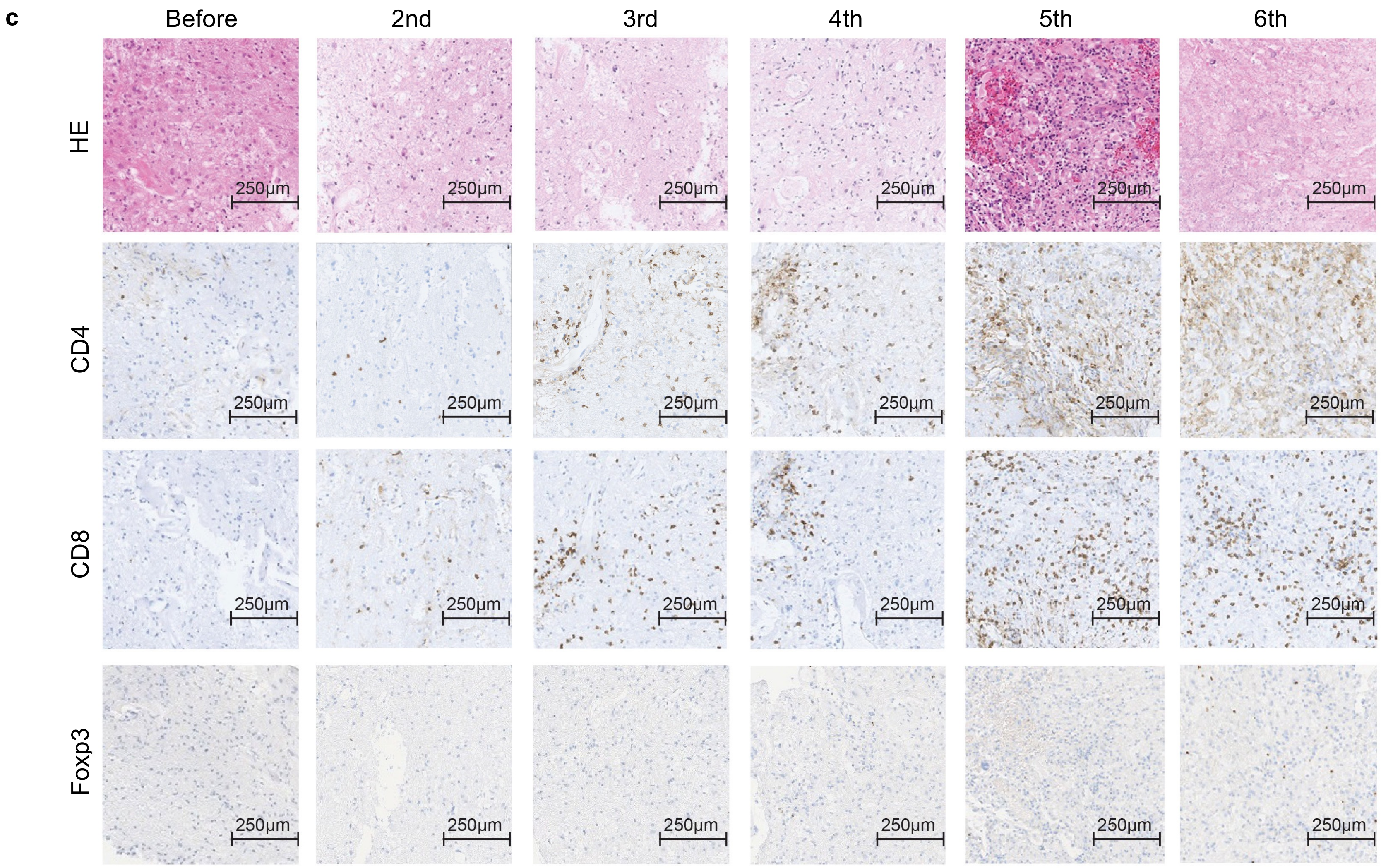

Supplement: Supplementary file 1 — Original and revised Fig. 2c [file 41591_2025_3619_MOESM1_ESM.pdf]
